# Xanthones with Potential Anti-Inflammatory and Anti-HIV Effects from the Stems and Leaves of *Cratoxylum cochinchinense*

**DOI:** 10.3390/molecules28166050

**Published:** 2023-08-14

**Authors:** Yong Zhang, Jia-Ming Guo, Ming-Ming Zhang, Ran Wang, Chai-Huan Liang, Yi-Meng Zhao, Ya-Yuan Deng, Yan-Ping Liu, Yan-Hui Fu

**Affiliations:** 1 Key Laboratory of Tropical Medicinal Resource Chemistry of Ministry of Education, Hainan Normal University, Haikou 571158, China; 2 Key Laboratory of Research and Development of Tropical Fruit and Vegetable of Haikou City, Hainan Normal University, Haikou 571158, China; 3 Key Laboratory of Southern Medicinal Plants Resources of Haikou City, Hainan Normal University, Haikou 571158, China; 4 Key Laboratory of Tropical Medicinal Plants Chemistry of Hainan Province, Hainan Normal University, Haikou 571158, China

**Keywords:** *Cratoxylum cochinchinense*, xanthones, cratocochinones A–D (**1**–**4**), anti-inflammatory effects, anti-HIV-1 effects

## Abstract

Four new xanthones, cratocochinones A–D (**1**–**4**), together with eight known analogues (**5**–**12**), were isolated from the stems and leaves of *Cratoxylum cochinchinense*. The chemical structures of cratocochinones A–D (**1**–**4**) were elucidated by comprehensive spectroscopic analyses and the known compounds were identified by comparisons with the spectral data reported in the literature. All isolated compounds **1**–**12** were evaluated for their anti-inflammatory activities and anti-HIV-1 activities. Compounds **1**–**12** showed remarkable inhibitory activities on nitric oxide (NO) production induced by lipopolysaccharide in mouse macrophage RAW 264.7 cells in vitro, with IC_50_ values in the range of 0.86 ± 0.05 to 18.36 ± 0.21 µM. Meanwhile, compounds **1**–**12** exhibited significant anti-HIV-1 activities with EC_50_ which ranged from 0.22 to 11.23 µM. These findings indicate that the discoveries of these xanthones, isolated from the stems and leaves of *C. cochinchinense,* showing significant anti-inflammatory and anti-HIV-1 effects could be of great importance to the research and development of new natural anti-inflammatory and anti-HIV agents.

## 1. Introduction

The genus *Cratoxylum* belonging to the Hypericaceae family consists of approximately six species, which are distributed in India, Myanmar, Thailand, through the Indo-China Peninsula, the south of China to Malaysia, Indonesia, and the Philippines, all in the south of 24 degrees north latitude. There are two species and one subspecies in China, mainly growing in Guangdong, Guangxi, and Yunnan provinces or regions [1]. Previous chemical investigations on the plants from the genus *Cratoxylum* have caused the isolation and identification of a variety of natural products including xanthones [2,3,4,5,6,7,8,9,10,11,12,13,14,15], triterpenoids [2], tocotrienols [2], bisanthraquinones [3], phloroglucinol benzophenones [15], and anthraquinobenzophenones [3], which display various biological activities, such as anti-tumor [3,6,8,9,10,14], anti-inflammatory [10,15], anti-bacterial [8,12], anti-oxidant [7,12], anti-malarial [6,9], anti-HIV [4], *α*-glucosidase inhibitory [15], vascular protective [7], retinoid X receptor*-α* transcriptional [11], protein tyrosine phosphatase 1B inhibitory [13], and NF-*κ*B inhibitory activities [10]. Among the genus *Cratoxylum*, *C. cochinchinense* (Lour.) Blume, a deciduous shrub or tree, is widely distributed in Hainan, Guangdong, Yunnan, and Guangxi provinces or regions in Southern China. The roots, barks, and tender leaves of *C. cochinchinense* are often used as a folk medicine by holding the effects of clearing heat and detoxifying, dispelling dampness and stagnation, as well as removing blood stasis and swelling [1]. Our preliminary experimental results revealed that the 85% ethanol extract of the stems and leaves of *C. cochinchinense* showed notable inhibitory effect against nitric oxide (NO) production induced by lipopolysaccharide in mouse macrophage RAW 264.7 cells with an IC_50_ value of 9.68 ± 0.12 μg/mL in vitro, as well as anti-HIV-1 reverse transcriptase (RT) effect with an EC_50_ value of 7.19 μg/mL. To fully explore the enormous potential of Hainan’s unique tropical medicinal plants and tropical fruits in the prevention and treatment of major human diseases, in the course of our continuing investigation of biologically active and structurally diverse natural products from the tropical medicinal plants and tropical fruits [16,17,18,19,20,21,22,23,24], a phytochemical study on the stems and leaves of *C. cochinchinense* was thus undertaken and led to the isolation and characterization of four new xanthones, cratocochinones A–D (**1**–**4**),which are a kind of rare natural product with extensive biological activities [2,3,4,5,6,7,8,9,10,11,12,13,14,15], alongside eight known analogues **5**–**12**. Their chemical structures were determined on the basis of comprehensive spectral analyses. Furthermore, to fully explore the enormous potential of these isolated xanthones in the development of anti-inflammatory and anti HIV drugs, all isolated xanthones **1**–**12** were evaluated for their anti-inflammatory and anti-HIV activities in vitro. Herein, the isolation, structure elucidation, anti-inflammatory effects, as well as anti-HIV activities of these isolated xanthones **1**–**12** will be reported.

## 2. Results and Discussion

### 2.1. Phytochemical Investigation

**Figure 1:**
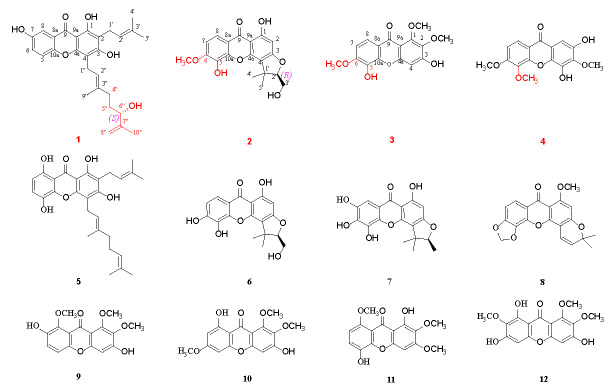
Chemical structures of compounds **1**–**12** isolated from *C. cochinchinense*.

The stems and leaves of *C. cochinchinense* were extracted by means of 85% ethanol and then suspended in purified water and extracted successively using petroleum ether and ethyl acetate. The ethyl acetate extract was repeatedly subjected to silica gel column chromatography (CC), Sephadex LH-20 gel CC, ODS gel CC, as well as *semi*-preparative HPLC, to afford four new xanthones **1**–**4**, along with eight know analogues **5**–**12**, as shown in Figure 1.

**Figure 2:**
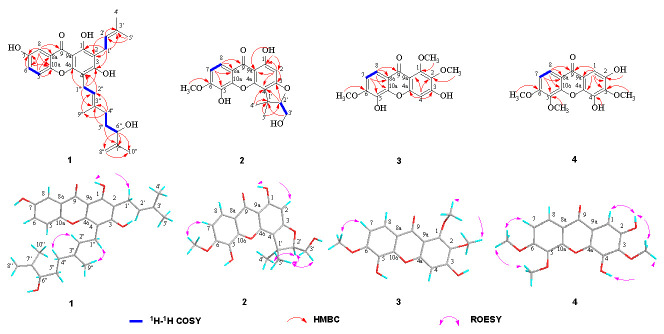
Selected 2D NMR correlations for cratocochinones A–D (**1**–**4**).

Cratocochinone A (**1**) was isolated as a pale white amorphous powder. The molecular formula of **1** was established as C_28_H_32_O_6_ on the basis of its HRESIMS (*m/z* 465.2276 [M + H]^+^, calculated 465.2272), requiring 13 indices of hydrogen deficiency. Its IR spectrum showed IR absorption bands at 3358, 3031, 2932, 1658, 1607, 1571, 1482, and 1379 cm^−1^, indicating the presence of a hydroxyl group, conjugated carbonyl group, benzene ring, double bond, and methyl group functionalities, respectively. Its UV spectrum displayed UV absorption bands at 231, 268, 318, and 377 nm, which were characteristic of xanthones [25]. The ^13^C-NMR and DEPT data of **1** (as shown in Table 1) revealed the presence of twenty-eight carbons, including nineteen sp^2^ carbons, one sp^3^ methine, four sp^3^ methylenes, and four methyls. Furthermore, the nineteen sp^2^ carbons were assigned with one to xanthone group, one to two tri-substituted double bonds, and one to terminal double bond. The above spectral data suggest that the chemical structure of **1** was similar to that of cochinchinone A [25], except that the 2-methylbut-2-ene group at C-4″ in cochinchinone A is substituted by a 3-methylbut-3-en-2-ol group in **1**, which was confirmed by the HMBC correlations of H-4″ to C-2″ (*δ*_C_ 122.5), C-3″ (*δ*_C_ 136.7) and C-9″ (*δ*_C_ 16.2), H-5″ to C-3″ and C-7″ (*δ*_C_ 147.1), H-6″ to C-7″, C-8″ (*δ*_C_ 111.2), and C-10″ (*δ*_C_ 17.9). The planar structure of **1** was unambiguously confirmed via the comprehensive analyses of its 2D NMR (HSQC, HMBC, ^1^H-^1^H COSY, and ROESY) spectra as shown in Figure 2. Furthermore, the observation of a strong cross-peak in the ROESY spectrum between H_2_-1″ and H_3_-9″ permitted assignment of the orientation of the olefinic bond between H-2″ and H-3″ as *E*. In addition, in view of cratocochinone (**1**) with a specific rotation of ${\left[\alpha \right]}_{D}^{28}$^−^18.6 (*c* 0.11, CH_3_OH) only containing a chiral center at C-6″, the absolute configuration at C-6″ in **1** was assigned to be *S* by comparison of its specific rotation with that of garcihombronone B, ${\left[\alpha \right]}_{D}^{26}$−16.2 (*c* 0.29, CH_3_OH) [26]. Accordingly, the chemical structure of **1** was determined as depicted in Figure 1.

Cratocochinone B (**2**) was separated as a pale white amorphous powder. Its molecular formula was determined as C_19_H_18_O_7_ on the basis of its HRESIMS (*m/z* 359.1128 [M + H]^+^, calculated 359.1126), requiring 11 degrees of unsaturation. Its IR spectrum exhibited IR absorptions at 3356, 3032, 2931, 1659, 1608, 1558, 1481, and 1382 cm^−1^, indicating the presence of s hydroxyl group, conjugated carbonyl group, benzene ring, double bond, and methyl group functionalities, respectively. The UV spectrum of **2** displayed UV absorptions at 229, 268, 315, and 375 nm, which were characteristic of xanthones [11]. The ^13^C-NMR and DEPT data of **2** (as shown in Table 1) suggest the presence of nineteen carbons, including thirteen sp^2^ carbons, one sp^3^ quaternary carbon, one sp^3^ methine, one sp^3^ methylene, and three methyls. Furthermore, the thirteen sp^2^ carbons were assigned to one xanthone group. Since 1 xanthone ring group accounted for ten out of eleven degrees of unsaturation, the remaining one degree of unsaturation was assumed for the presence of another ring system in **2**. The above spectral data suggested that the chemical structure of 2 was similar to that of cochinchinone P [11], except that the hydroxyl group at C-6 in cochinchinone P was substituted by a methoxy group in **2**, which was verified by the presence of the methoxy group resonating at *δ*_H_ 3.86 (3H, s, 6-OCH_3_), and *δ*_C_ 60.5, which was further confirmed by the HMBC correlations of H-7, H-8, and 6-OCH_3_ to C-6 (*δ*_C_ 134.6), as well as the ROESY correlation of 6-OCH_3_ and H-7. Furthermore, the planar structure of **2** was unambiguously confirmed by the comprehensive analyses of its 2D NMR (HSQC, HMBC, ^1^H-^1^H COSY, and ROESY) spectra as shown in Figure 2. Additionally, in view of the fact that 2 holds a specific rotation of ${\left[\alpha \right]}_{D}^{28}$+58.3 (c 0.13, CH_3_OH) only possessing a chiral carbon at C-2′, therefore, the absolute configuration of **2** at C-2′ could be assigned to be 2′*R*, which is the same as that of pruniflorone M, whose absolute configuration had been unquestionably determined by the X-ray single crystal diffraction method, on the basis of their identical chiral structures and similar rotation values [27,28]. Thus, the chemical structure of **2** was determined as depicted in Figure 1.

Cratocochinone C (**3**) is obtained as a pale-yellow amorphous powder. The molecular formula of **3** was acquired as C_16_H_14_O_7_ according to its HRESIMS with *m/z* 319.0818 (calculated for C_16_H_15_O_7_ [M + H]^+^, 319.0813) indicating 10 degrees of unsaturation. The IR absorptions at 3356, 3030, 2932, 1658, 1607, 1568, and 1483 cm^−1^ showed the presence of a hydroxyl group, ketocarbonyl group, and benzene ring group. The UV absorption bands at 227, 267, 316, and 378 nm were characteristic of a xanthone derivative [29]. The ^13^C-NMR and DEPT data (as shown in Table 2) revealed the presence of sixteen carbon atoms, including thirteen sp^2^ carbon atoms, and three methyls. In addition, the thirteen sp^2^ carbon atoms were attributable to one xanthone ring group. The above data revealed that the chemical structure of **3** was similar to that of calophymembranol C [29], except that the methyl group located at C-5 and the hydroxyl group located at C-6 in calophymembranol C were substituted by a methyl group and a hydroxyl group in 3, respectively, which was supported the HMBC correlations of H-7, H-8, and 6-OCH_3_ to C-6 (*δ*_C_ 145.2), along with the ROSEY correlation of H-7 with 6-OCH_3_. Comprehensive analysis of its 2D NMR (HSQC, HMBC, ^1^H-^1^H COSY, and ROESY) spectra confirmed the chemical structure of **3** as shown in Figure 2. Hence, the chemical structure of **3** was established as shown in Figure 1.

Cratocochinone D (**4**) holds a molecular formula of C_16_H_14_O_7_, which was defined by its HRESIMS spectrum exhibiting a [M + H]^+^ ion peak at *m/z* 319.0816 (calculated for C_16_H_15_O_7_ [M + H]^+^, 319.0813) indicating 10 indices of hydrogen deficiency. The ^13^C NMR and DEPT data of **4** revealed the presence of sixteen carbon atoms, including thirteen sp^2^ carbon atoms, and three methyl groups. In addition, the thirteen sp^2^ carbon atoms were attributable to one keto carbonyl group and two benzene ring groups. The above data revealed that the chemical structure of **4** was similar to that of 2,4-dihydroxy-3,6-dimethoxy-9H-xanthen-9-one [30]. Comparison of the NMR data of **4** (as shown in Table 2) with those of 2,4-dihydroxy-3,6-dimethoxy-9H-xanthen-9-one suggested that both compounds shared the same basic skeleton, but the molecular weight of **4** is larger than that of 2,4-dihydroxy-3,6-dimethoxy-9H-xanthen-9-one by 30 units, namely, the hydrogen atom at C-5 in 2,4-dihydroxy-3,6-dimethoxy-9H-xanthen-9-one was replaced by a methoxy group resonating at *δ*_H_ 3.79 (3H, s) and *δ*_C_ 60.9 in **4**. The above elucidation was supported by the HMBC correlations of the methoxy hydrogens resonating at *δ*_H_ 3.79 (3H, s) to C-5 (*δ*_C_ 144.9), as well as the ROSEY correlations of 6-OCH_3_, and the methoxy group resonating at *δ*_H_ 3.85 (3H, s) with 5-OCH_3_ and H-7. Comprehensive analysis of 2D NMR (HSQC, HMBC, ^1^H-^1^H COSY, and ROSEY) spectra confirmed the chemical structure of **4** as shown in Figure 2. Thus, the chemical structure of **4** was established as shown in Figure 1.

In addition to the four new xanthones **1**–**4**, eight known xanthones **5**–**12** were isolated and identified as 1,3,5,8-tetrahydroxy-2-(3-methybut-2-enyl)-4-(3,7-dimethylocta-2,6-dienyl) xanthone (**5**) [31], cochinchinone P (**6**) [27], 2-deprenyl-7-hydroxy-rheediaxanthone (**7**) [32], musaxanthone (**8**) [33], 3,7-dihydroxy-1,2,8-trimethoxy-9H-xanthen-9-one (**9**) [34], 3,8-dihydroxy-1,2,6-trimethoxy-9H-xanthen-9-one (**10**) [35], 1,5-dihydroxy-2,3,8-trimethoxy-9H-xanthen-9-one (**11**) [12], and 1,3,6-trihydroxy-2,7,8-trimethoxy-9H-xanthen-9-one (**12**) [36] by comparing their experimental spectral data with the reported spectra data in the literature.

### 2.2. Anti-Inflammatory Activity

All isolated xanthones **1**–**12** were evaluated for their anti-inflammatory effects by examining their inhibitory activities against NO production in vitro. Meanwhile, the MTT assay was used for evaluating the cytotoxic activities of those isolated xanthones against mouse macrophage RAW 264.7 cells. As a result, all isolated xanthones **1**–**12** showed notable inhibitory activities against NO production, thereby holding IC_50_ values in range of 0.86 ± 0.05 to 18.36 ± 0.21 μM (as shown in Table 3) which are comparable to that of the positive control (hydrocortisone). No cytotoxicity was observed in the macrophage RAW 264.7 cells treated by those isolated xanthones **1**–**12** (cell viability > 90%).

### 2.3. Anti-HIV-1 Activity

All isolated xanthones **1**–**12** were assessed for their anti-HIV reverse transcriptase (RT) effects according to the inhibition assay for the cytopathic activities of HIV-1 (EC_50_) as well as the cytotoxic activities assay against C8166 cell line (CC_50_) according to MTT methods. Xanthones **1**–**12** exhibited notable anti-HIV affects with EC_50_ values in the range from 0.22 to 11.23 µM (as displayed in Table 4). No cytotoxicity was observed against the C8166 cell line treated with the isolated xanthones **1**–**12** (CC_50_ > 200.00 µM).

## 3. Experimental Section

### 3.1. General Experiment Procedure

The optical rotations of compounds **1**, **2**, **6,** and **7** were recorded by means of a JASCO P-1020 digital polarimeter (Jasco Corp., Tokyo, Japan). The UV spectra of new compounds **1**–**4** were recorded though a PharmaSpec UV-1700 spectrophotometer (Shimadzu Corp., Kyoto, Japan). The IR spectra of new compounds **1**–**4** were measured using a Bio-Rad FTS-135 spectrophotometer (KBr) (Bio-Rad Laboratories, Richmond, CA, USA). The HRESIMS spectra of compounds **1**–**12** were recorded though an Acquity UPLC-Q-TOF Micro-mass spectrometer (Waters Corp., Milford, MA, USA); The NMR spectra of compounds **1**–**12** were measured by means of a Bruker Avance III-400 MHz NMR spectrometer (Bruker Corp., Karlsruhe, Germany). The semi-preparative HPLC was performed on a Thermo Fisher UltiMate 3000 LC series (Thermo Fisher Scientific Inc., Waltham, MA, USA) equipped with a MWD detector using an Agilent Eclipse XDB-C18 column (5 μm, 250 × 9.4 mm). Silica gel (100−200, 200−300 mesh) was used for performing open column chromatography (CC), and was purchased from Anhui Liangchen Silicon Material Co., Ltd. (Liuan, China). ODS gel (50 μm) was applied to fulfill medium-pressure CC, and was a product of YMC (YMC Co., Ltd., Kyoto, Japan). Precoated silica gel plates (GF-254) were utilized for implementing thin-layer chromatography (TLC), and were purchased from Anhui Liangchen Silicon Material Co., Ltd. (Liuan, China).

### 3.2. Plant Material

The stems and leaves of *C. cochinchinense* were collected from Bawangling Nature Reserve, Hainan Province, China, in June 2020, and identified by Prof. Qiong-Xin Zhong, College of Life Science, Hainan Normal University. A voucher specimen (No. CRCO20200608) has been deposited at the Key Laboratory of Tropical Medicinal Resource Chemistry of Ministry of Education, Hainan Normal University.

### 3.3. Extraction and Isolation

The air-dried stems and leaves of *C. cochinchinense* (22.3 kg) were powdered and then extracted seven times by means of 85% ethanol (45.8 L) at room temperature (28−35 °C), each time for two days. The ethanol solvent was combined and condensed to yield a crude extract under reduced pressure. The crude extract was further suspended in purified water (23.0 L) and was then successively extracted five times with petroleum ether (23.0 L) and six times with ethyl acetate (23.0 L), to obtain the petroleum ether extract (687.3 g) and the ethyl acetate extract. The ethyl acetate extract (1028.6 g) was subjected to a silica gel CC eluted by chloroform/acetone (2:98 to 40:60, *v*/*v*) to yield eight fractions (Fr.1–Fr.8). Fr.2 (60.9 g) was subjected to an ODS gel medium-pressure CC (methanol/purified water, 45:55 to 100:0) to give seven subfractions 2A−2G. Fr. 2B (5.3 g) was purified using a Sephadex LH-20 gel CC (methanol) and then was prepared by means of a semi-preparative HPLC (acetonitrile/purified water, 68:32; 5.2 mL/min, tR 28.9, 36.8 and 41.2 min) to yield **1** (21.5 mg), **5** (9.7 mg), and **10** (36.7 mg). Fr. 2C (6.8 g) was purified by a Sephadex LH-20 gel CC (methanol) and then was prepared using a *semi*-preparative HPLC (methanol/purified water, 76:24; 4.2 mL/min, tR 19.8, 37.2 and 46.8 min) to afford **2** (42.2 mg), **6** (53.1 mg) and **9** (7.3 mg). Fr.3 (52.8 g) was subjected to an ODS gel medium-pressure CC (methanol/purified water, 35:65 to 100:0) to yield eight subfractions 3A−3H. Fr. 3B (7.1 g) was purified using a Sephadex LH-20 gel CC (methanol) and then was prepared by means of a semi-preparative HPLC (acetonitrile/purified water, 58:42; 5.8 mL/min, t_R_ 28.3, 35.2 and 39.8 min) to give **3** (24.6 mg), **4** (75.3 mg), and **11** (9.3 mg). Fr. 3C (4.3 g) was purified though a Sephadex LH-20 gel CC (methanol) and then was prepared using a semi-preparative HPLC (methanol/purified water, 63:37; 3.9 mL/min, t_R_ 31.8, 38.7 and 46.2 min) to produce **7** (11.3 mg), **8** (16.8 mg), and **12** (108.9 mg).

*Cratocochinone A* (**1**): Pale yellow amorphous powder; ${\left[\alpha \right]}_{D}^{28}$−18.6 (*c* 0.11, CH_3_OH); UV (CH_3_OH) λ_max_ (log *ε*) 231 (4.60), 268 (4.78), 318 (4.06), and 377 (3.68) nm; IR (KBr) *v*_max_ 3358, 3031, 2932, 1658, 1607, 1571, 1482, 1379, 1258, 1147, 1063, 962, 859, and 726 cm^–1^; ^1^H and ^13^C NMR data (as shown in Table 1); HRESIMS *m/z* 465.2276 [M + H]^+^ (calculated for C_28_H_33_O_6_, 465.2272).

*Cratocochinone B* (**2**): Pale yellow amorphous powder; ${\left[\alpha \right]}_{D}^{28}$+58.3 (c 0.13, CH_3_OH); UV (CH_3_OH) λ_max_ (log *ε*) 229 (4.26), 268 (4.53), 315 (3.89), and 375 (3.52) nm; IR (KBr) *v*_max_ 3356, 3032, 2931, 1659, 1608, 1558, 1481, 1382, 1258, 1147, 1068, 961, 859, and 727 cm^–1^; ^1^H and ^13^C NMR data (as shown in Table 1); HRESIMS *m/z* 359.1128 [M + H]^+^ (calculated for C_19_H_19_O_7_, 359.1126).

*Cratocochinone C* (**3**): Pale yellow amorphous powder; UV (CH_3_OH) λ_max_ (log *ε*) 227 (4.28), 267 (4.59), 316 (4.02), and 378 (3.73) nm; IR (KBr) *v*_max_ 3356, 3030, 2932, 1658, 1607, 1568, 1483, 1379, 1257, 1148, 1069, 961, 867, and 725 cm^–1^; ^1^H and ^13^C NMR data (as shown in Table 2); HRESIMS *m/z* 319.0818 [M + H]^+^ (calculated for C_16_H_15_O_7_, 319.0813).

*Cratocochinone D* (**4**): Pale yellow amorphous powder; UV (CH_3_OH) λ_max_ (log ε) 228 (4.46), 268 (4.82), 315 (3.89), and 376 (3.68) nm; IR (KBr) *v*_max_ 3363, 3027, 2930, 1659, 1611, 1568, 1481, 1381, 1259, 1148, 1069, 961, 868, and 730 cm^–1^; ^1^H and ^13^C NMR data (as shown in Table 2); HRESIMS *m/z* 319.0816 [M + H]^+^ (calculated for C_16_H_15_O_7_, 319.0813).

### 3.4. Anti-Inflammatory Bioassays

All isolated xanthones **1**–**12** were appraised for their anti-inflammatory activities on the basis of measuring the inhibitory effects against NO production induced by lipopolysaccharide in mouse macrophage RAW 264.7 cells in vitro. The protocol for this assay is described in detail in the Supplementary Material of our current article [37,38,39], in which hydrocortisone was utilized as the positive control.

### 3.5. Anti-HIV-1 Activity Bioassays

All isolated xanthones **1**–**12** were assessed for their anti-HIV-1 reverse transcriptase (RT) effects in vitro on the basis of the inhibition assay for the cytopathic effects of HIV-1 (EC_50_), in light of the protocol which is described in detail in the Supplementary Material of our current article [40,41,42], in which AZT (3′-azido-3′-deoxythymidine) was adopted as the positive control. All isolated xanthones **1**–**12** and the positive control (AZT) were also evaluated for their cytotoxic activities against C8166 cells (CC_50_) on the basis of the MTT method [40,41,42].

## 4. Conclusions

In this investigation, the chemical study on the stems and leaves of *C. cochinchinense* was conducted and led to the isolation and identification of four new xanthones, cratocochinones A–D (**1**–**4**), together with eight known xanthones **5**–**12**. The discovery of four new xanthones **1**–**4** is not only a further addition to diverse and highly aromatic array of xanthones, but also, their presence as characteristic markers might be helpful in chemotaxonomical classifications. All isolated xanthones **1**–**12** were also investigated for their anti-inflammatory effects and anti-HIV-1 activities, and were proven to be very powerful. In particular, among these isolated compounds, compounds **1**–**3** and **11** displayed stronger inhibitory effects against NO production with IC_50_ values ranging from 0.86 ± 0.05 to 3.16 ± 0.18 μM, which were below that of hydrocortisone. These remarkable inhibitory activities against nitric oxide (NO) production of xanthones **1**–**12** might be used as an explanation of the folk applications of the stems and leaves of *C. cochinchinense*, which are applied as an anti-inflammatory ethnic drug in China. These findings also indicate that these isolated xanthones, isolated from the stems and leaves of *C. cochinchinense* with notable inhibitory activities on nitric oxide (NO) production and anti-HIV-1 effects, could be used for the research and development of new anti-inflammatory and anti-HIV agents.

## Figures and Tables

**Table 1:** ^1^H and ^13^C NMR data of cratocochinones A (**1**) and B (**2**).

Position	Cratocochinone A (1)		Cratocochinone B (2)	
	*δ*_H_ ^a^	*δ*_C_ ^b^	*δ*_H_ ^c^	*δ*_C_ ^d^
1		158.1 s		163.4 s
2		108.8 s	6.22 (1H, s)	93.0 d
3		160.9 s		165.1 s
4		105.1 s		112.6 s
5	7.26 (1H, d, *J* = 8.0 Hz)	118.8 d		150.4 s
6	7.18 (1H, dd, *J* = 8.0, 1.8 Hz)	124.1 d		134.6 s
7		150.2 s	6.91 (1H, d, *J* = 8.8 Hz)	115.3 d
8	7.48 (1H, d, *J* = 1.8 Hz)	108.6 d	7.69 (1H, d, *J* = 8.8 Hz)	121.0 d
9		180.8 s		179.1 s
4a		153.0 s		152.0 s
8a		120.4 s		111.1 s
9a		103.1 s		102.2 s
10a		152.5 s		160.3 s
1′	3.42 (2H, d, *J* = 6.8 Hz)	21.5 t		42.6 s
2′	5.28 (1H, d, *J* = 6.8 Hz)	121.5 d	4.44 (1H, dd, *J* = 6.8, 4.6 Hz)	94.2 d
3′		135.5 s	3.79 (1H, dd, *J* = 12.1, 4.6 Hz)	59.6 t
			3.74 (1H, dd, *J* = 12.1, 6.8 Hz)	
4′	1.77 (3H, s)	25.8 q	1.62 (3H, s)	26.3 q
5′	1.84 (3H, s)	17.6 q	1.34 (3H, s)	20.9 q
1″	3.51 (2H, d, *J* = 6.8 Hz)	21.7 t		
2″	5.37 (1H, d, *J* = 6.8 Hz)	122.5 d		
3″		136.7 s		
4″	2.11 (2H, m)	35.9 t		
5″	1.70 (2H, m)	32.8 t		
6″	4.08 (1H, d, *J* = 6.8 Hz)	75.9 d		
7″		147.1 s		
8″	4.90 (1H, s)	111.2 t		
	4.81 (1H, s)			
9″	1.89 (3H, s)	16.2 q		
10″	1.70 (3H, s)	17.9 q		
1-OH	13.02 (1H, s)		13.52 (1H, s)	
6-OCH_3_			3.86 (3H, s)	60.5 q

^a^ Measured at 400 MHz in CDCl_3_. ^b^ Measured at 100 MHz in CDCl_3_.^c^ Measured at 400 MHz in DMSO-*d*_6_. ^d^ Measured at 100 MHz in DMSO-*d*_6_.

**Table 2:** ^1^H and ^13^C-NMR data of cratocochinones C (**3**) and D (**4**).

Position	Cratocochinone C (3)		Cratocochinone D (4)	
	*δ*_H_ ^a^	*δ*_C_ ^b^	*δ*_H_ ^a^	*δ*_C_ ^b^
1		152.0 s	6.99 (1H, s)	94.0 d
2		140.8 s		149.8 s
3		166.4 s		141.9 s
4	6.23 (1H, s)	99.5 d		140.1 s
5		148.5 s		144.9 s
6		145.2 s		149.7 s
7	7.16 (1H, d, *J* = 8.8 Hz)	121.4 d	7.37 (1H, d, *J* = 9.1 Hz)	123.5 d
8	6.98 (1H, d, *J* = 8.8 Hz)	112.1 d	7.24 (1H, d, *J* = 9.1 Hz)	113.6 d
9		173.1 s		174.8 s
4a		154.4 s		141.8 s
8a		117.1 s		115.7 s
9a		104.8 s		117.1 s
10a		146.3 s		146.6 s
1-OCH_3_	3.75 (3H, s)	61.1 q		
2-OH			10.89 (1H, s)	
2-OCH_3_	3.62 (3H, s)	60.0 q		
3-OCH_3_			3.82 (3H, s)	60.3 q
4-OH			9.47 (1H, s)	
5-OCH_3_			3.79 (3H, s)	60.9 q
6-OCH_3_	3.72 (3H, s)	60.9q	3.85 (3H, s)	55.7 q

^a^ Measured at 400 MHz in DMSO-*d*_6_. ^b^ Measured at 100 MHz in DMSO-*d*_6_.

**Table 3:** Anti-inflammatory effects of compounds **1**–**12**.

Compound	IC_50_ (μM) ^a^	Compound	IC_50_ (μM) ^a^
1	1.29 ± 0.06	7	12.58 ± 0.12
2	2.17 ± 0.09	8	4.16 ± 0.08
3	0.86 ± 0.05	9	18.36 ± 0.21
4	9.42 ± 0.13	10	7.47 ± 0.11
5	6.47 ± 0.10	11	3.16 ± 0.09
6	8.35 ± 0.15	12	6.21 ± 0.08
Hydrocortisone ^b^	4.08 ± 0.08		

^a^ IC_50_ value was defined as 50% inhibitory concentration on NO production induced by lipopolysaccharide in mouse macrophage RAW 264.7 cells in vitro and expressed as the mean ± SD of triplicate determinations. ^b^ Positive control.

**Table 4:** Anti-HIV-1 effects of compounds **1**–**12**.

Compound	CC_50_ (µM) ^a^	EC_50_ (µM) ^b^	TI ^c^
1	>200.00	0.22	>909.09
2	>200.00	0.68	>294.12
3	>200.00	1.89	>105.82
4	>200.00	4.07	>49.14
5	>200.00	2.53	>79.05
6	>200.00	3.08	>64.94
7	>200.00	4.16	>48.08
8	>200.00	8.91	>22.45
9	>200.00	5.32	>37.59
10	>200.00	2.67	>74.91
11	>200.00	11.23	>17.81
12	>200.00	6.35	>31.50
AZT ^d^	4018.26	0.02078	193,371.51

^a^ CC_50_: 50% Cytotoxic concentration. ^b^ EC_50_: 50% Effective concentration. ^c^ TI (therapeutic index) = CC_50_/EC_50_. ^d^ AZT (3′-azido-3′-deoxythymidine) was used as a positive control.

## Data Availability

The authors confirm that the data supporting the findings of this study are available within the article or its Supplementary Materials.

## Supplementary Materials

The following supplementary materials can be downloaded at: https://www.mdpi.com/article/10.3390/molecules28166050/s1. Anti-inflammatory bioassays, anti-HIV-1 activity bioassays as well as 1D and 2D NMR spectra of new compounds **1**–**4** are available online as Supplementary Materials.

## Sample Availability

Samples of all isolated compounds **1**–**12** are available from the authors.
