# Supplementary material for: Xanthones with Potential Anti-Inflammatory and Anti-HIV Effects from the Stems and Leaves of Cratoxylum cochinchinense
Source: Molecules. 2023 Aug 14;28(16):6050. doi: 10.3390/molecules28166050 (PMC10458312; doi:10.3390/molecules28166050)
Supplement: Supplementary file 1 [file molecules-28-06050-s001.zip › molecules-2515850-supplementary.pdf]

Supplementary Material

Xanthones with Potential Anti-inflammatory and Anti-HIV Effects from the Stems and Leaves of *Cratoxylum cochinchinense*

Yong Zhang [1], Jia-Ming Guo [1], Ming-Ming Zhang [1], Ran Wang [1], Chai-Huan Liang [1], Yi-Meng Zhao [1], Ya-Yuan Deng [1], Yan-Ping Liu [1,2,3,4,*] and Yan-Hui Fu [1,2,3,4,*]

[1] Key Laboratory of Tropical Medicinal Resource Chemistry of Ministry of Education, Hainan Normal University, Haikou 571158, China

[2] Key Laboratory of Research and Development of Tropical Fruit and Vegetable of Haikou City, Hainan Normal University, Haikou 571158, China

[3] Key Laboratory of Southern Medicinal Plants Resources of Haikou City, Hainan Normal University, Haikou 571158, China

[4] Key Laboratory of Tropical Medicinal Plants Chemistry of Hainan Province, Hainan Normal University, Haikou 571158, China

* Correspondence: liuyanpinghs@163.com (Y.-P.L.); fuyanhui80@163.com (Y.-H.F.); Tel: +86-898-65889422. Fax: +86-898-65889422.

Contents of Supplementary Material

Anti-inflammatory bioassays

Anti-HIV-1 activity bioassays

Figure S1. ^{1}H NMR spectrum of cratocochinone A (**1**) in $CDCl_3$.

Figure S2. ^{13}C NMR spectrum of cratocochinone A (**1**) in $CDCl_3$.

Figure S3. HSQC spectrum of cratocochinone A (**1**) in $CDCl_3$.

Figure S4. HMBC spectrum of cratocochinone A (**1**) in $CDCl_3$.

Figure S5. ^{1}H-^{1}H COSY spectrum of cratocochinone A (**1**) in $CDCl_3$.

Figure S6. ROESY spectrum of cratocochinone A (**1**) in $CDCl_3$.

Figure S7. ^{1}H NMR spectrum of cratocochinone B (**2**) in DMSO-d_6.

Figure S8. ^{13}C NMR spectrum of cratocochinone B (**2**) in DMSO-d_6.

Figure S9. HSQC spectrum of cratocochinone B (**2**) in DMSO-d_6.

Figure S10. HMBC spectrum of cratocochinone B (**2**) in DMSO-d_6.

Figure S11. ^{1}H-^{1}H COSY spectrum of cratocochinone B (**2**) in DMSO-d_6.

Figure S12. ROESY spectrum of cratocochinone B (**2**) in DMSO-d_6.

Figure S13. ^{1}H NMR spectrum of cratocochinone C (**3**) in DMSO-d_6.

Figure S14. ^{13}C NMR spectrum of cratocochinone C (**3**) in DMSO-d_6.

Figure S15. HSQC spectrum of cratocochinone C (**3**) in DMSO-d_6.

Figure S16. HMBC spectrum of cratocochinone C (**3**) in DMSO-d_6.

Figure S17. ^{1}H-^{1}H COSY spectrum of cratocochinone C (**3**) in DMSO-d_6.

Figure S18. ^{1}H NMR spectrum of cratocochinone D (**4**) in DMSO-d_6.

Figure S19. ^{13}C NMR spectrum of cratocochinone D (**4**) in DMSO-d_6.

Figure S20. HSQC spectrum of cratocochinone D (**4**) in DMSO-d_6.

Figure S21. HMBC spectrum of cratocochinone D (**4**) in DMSO-d_6.

Figure S22. ^{1}H-^{1}H COSY spectrum of cratocochinone D (**4**) in DMSO-d_6.

Anti-inflammatory bioassays

The RAW 264.7 cells were incubated in RPMI 1640 medium containing 10% fetal bovine serum, 2.0 mmol/L glutamine, 100.0 U/mL penicillin, and 100.0 μg/mL streptomycin. Cell concentration was adjusted to 5×10^5 cells/mL, and 200.0 μL of cell suspension was seeded in each well of a 96-well plate. After 1 h incubation, cells were treated with LPS (1.0 μg/mL) and test samples were dissolved in DMSO at concentrations of 0.0625, 0.32, 1.6, 8.0 and 40.0 μM (final DMSO concentration 0.2%, *v*/*v*) for 24 h at 37 °C. A 100.0 μL sample of the culture supernatant was determined by the Griess reaction The Griess reagent (50.0 μL of 1% sulfanilamine in 5% H_3PO_4, and 50.0 μL of 0.1% *N*-1-naphthyl ethylenediamine dihydrochloride) was added to each well. After 10 min, the reaction products were colorimetrically quantitated at 540 nm using a microplate reader. The experiments were performed in triplicate. Hydrocortisone was used as a positive control; The cytotoxicity assay was performed using the MTT method in 96-well microplates. An MTT solution (200.0 μg/mL) was added after the 24 h treatment and then incubated for another 4 h at 37 °C. The reduced MTT-formazan was solubilized with 150.0 μL of DMSO, and the absorbance of the MTT-formazan solution at 570 nm was measured by an immunoreader. The percentage of suppression was calculated by comparing the absorbance of sample treated cells with that of nontreated cells.

Anti-HIV-1 activity bioassays

Cytotoxicity against C8166 cells (CC_{50}) was assessed using the MTT method, and anti-HIV-1 activity was evaluated by the inhibition assay for the cytopathic effects of HIV-1 (EC_{50}). Briefly, cells were seeded on a microtiter plate in the absence or presence of various concentrations of compounds in triplicate and incubated at 37 °C in a humid atmosphere of 5% CO_2 for three days. 20.0 μL MTT reagent (5.0 mg/mL in PBS) was added to each well, then incubated at 37 °C for 4 h, 50% DMF-20% SDS (100.0 μL) was added. After the formazan was dissolved completely, the plates were read on a Bio-Tek ELx 800 ELISA reader at 595 nm/630 nm (A595/630). The cytotoxic concentration that caused the reduction of viable cells by 50% (CC_{50}) was calculated from dose–response curve. In 100.0 μL various concentrations of compounds, C8166 cells (4×10^5/mL) were infected with virus (HIV-1IIIB) at a multiplicity of infection (M. O. I) of 0.06. The final volume per well was 200.0 μL. Control assays were performed without the testing compounds in HIV-1IIIB infected and uninfected cultures. AZT (3′-azido-3′-deoxythymidine) was used as a positive control. After three days of culture, the cytopathic effect (CPE) was measured by counting the number of syncytia (multinucleated giant cell). Percentage inhibition of syncytia formation was calculated and 50% effective concentration (EC_{50}) was calculated. The therapeutic index (TI) was calculated from the ratio of CC_{50}/EC_{50}.

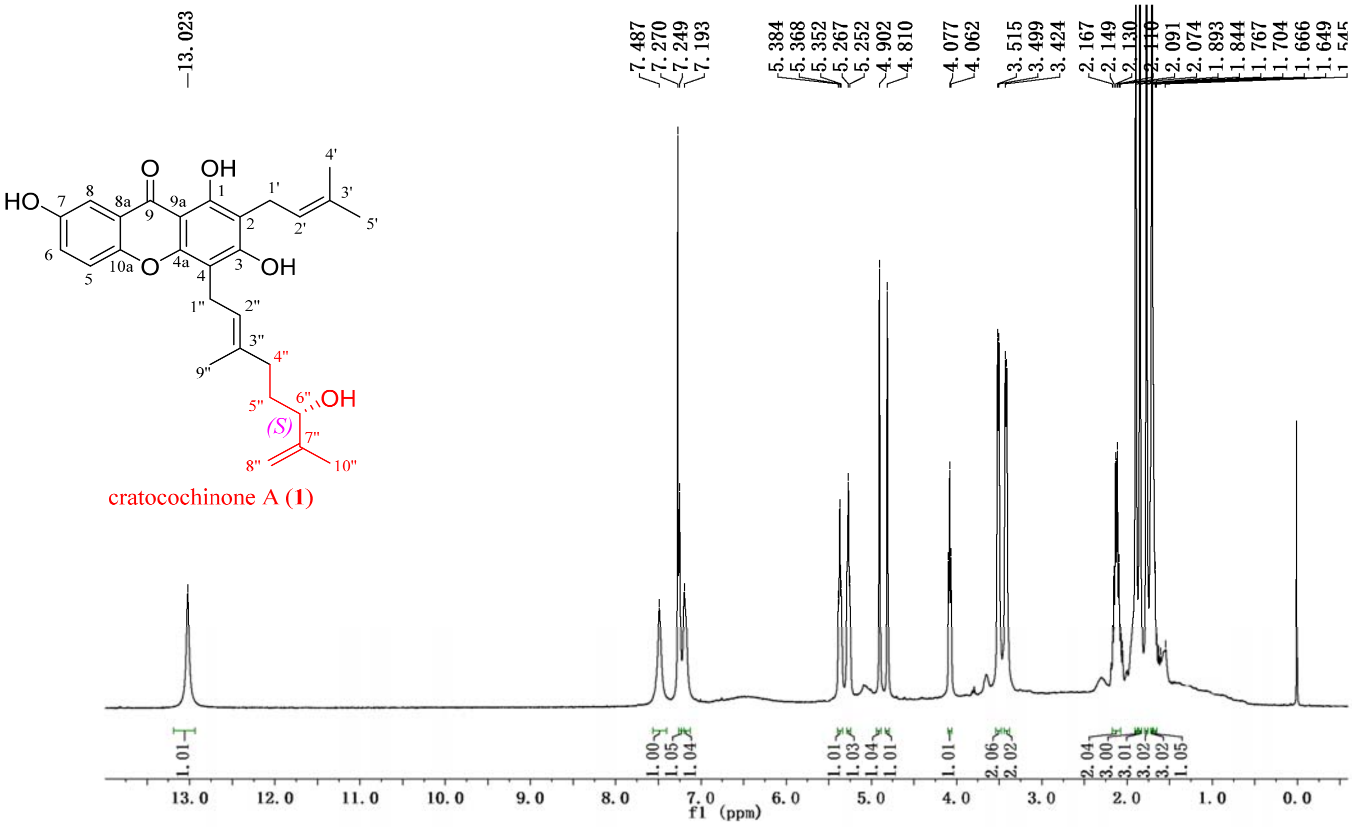

Figure S1. ^{1}H NMR spectrum of cratocochinone A (**1**) in $CDCl_3$.

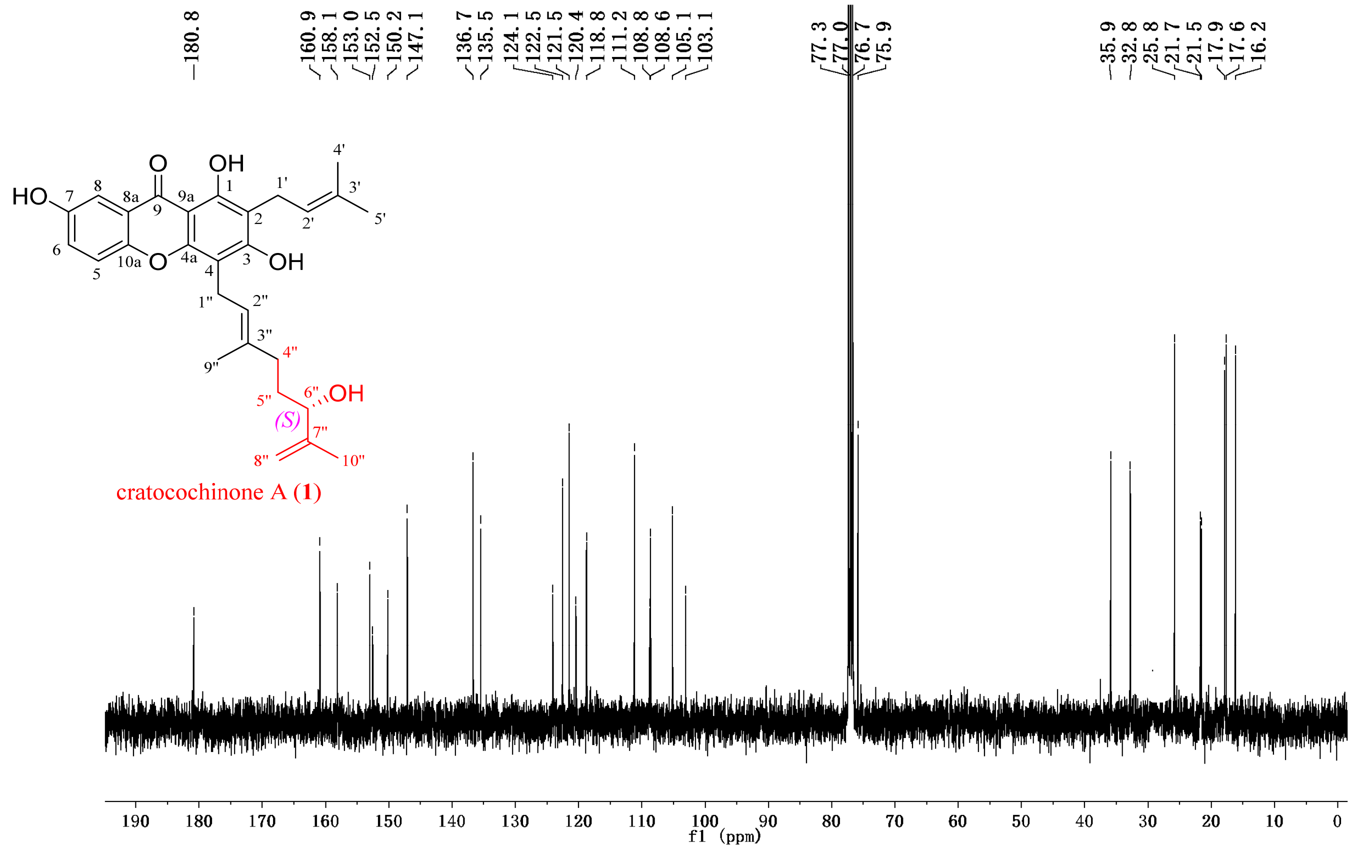

Figure S2. ^{13}C NMR spectrum of cratocochinone A (**1**) in $CDCl_3$.

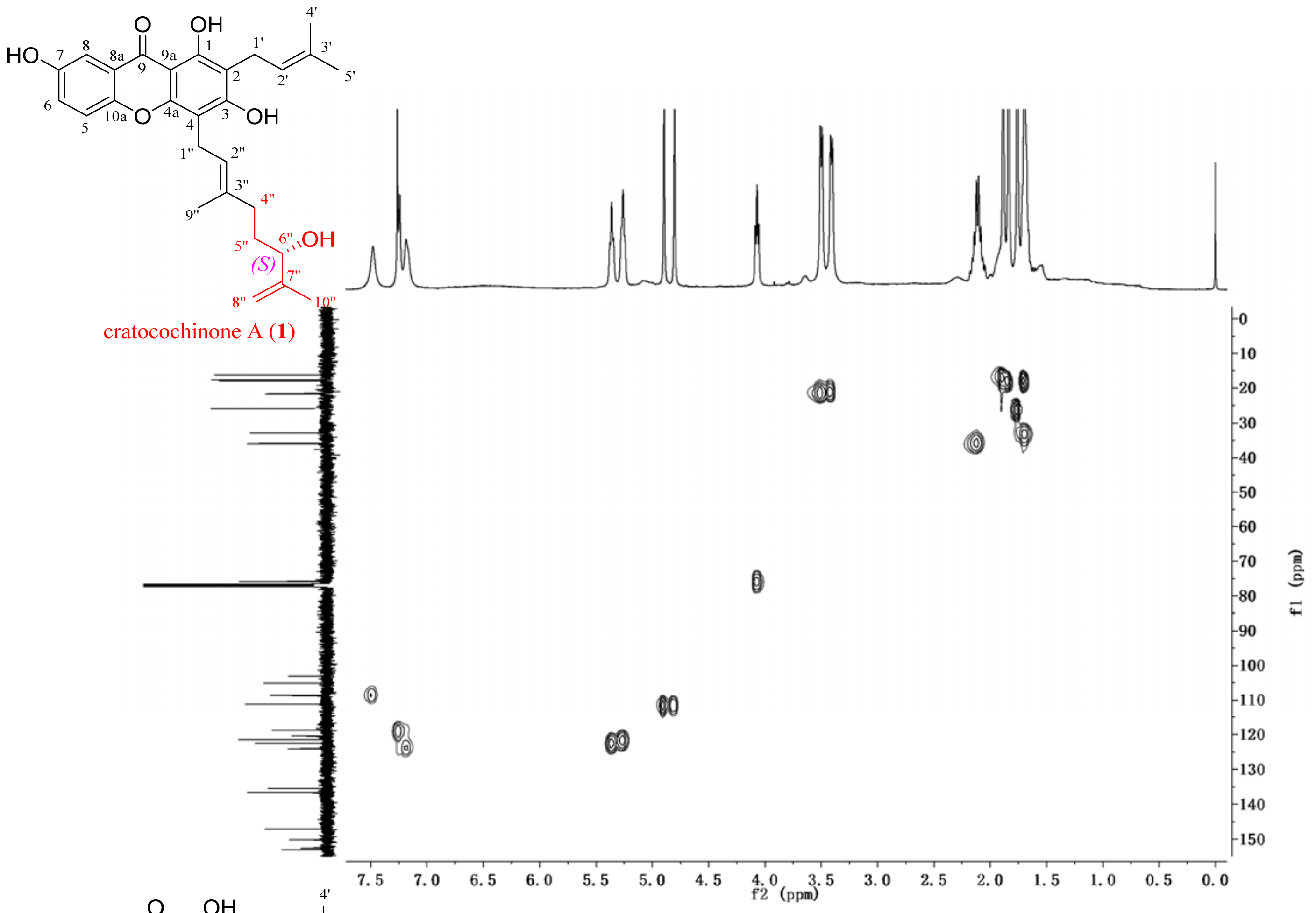

Figure S3. HSQC spectrum of cratocochinone A (**1**) in $CDCl_3$.

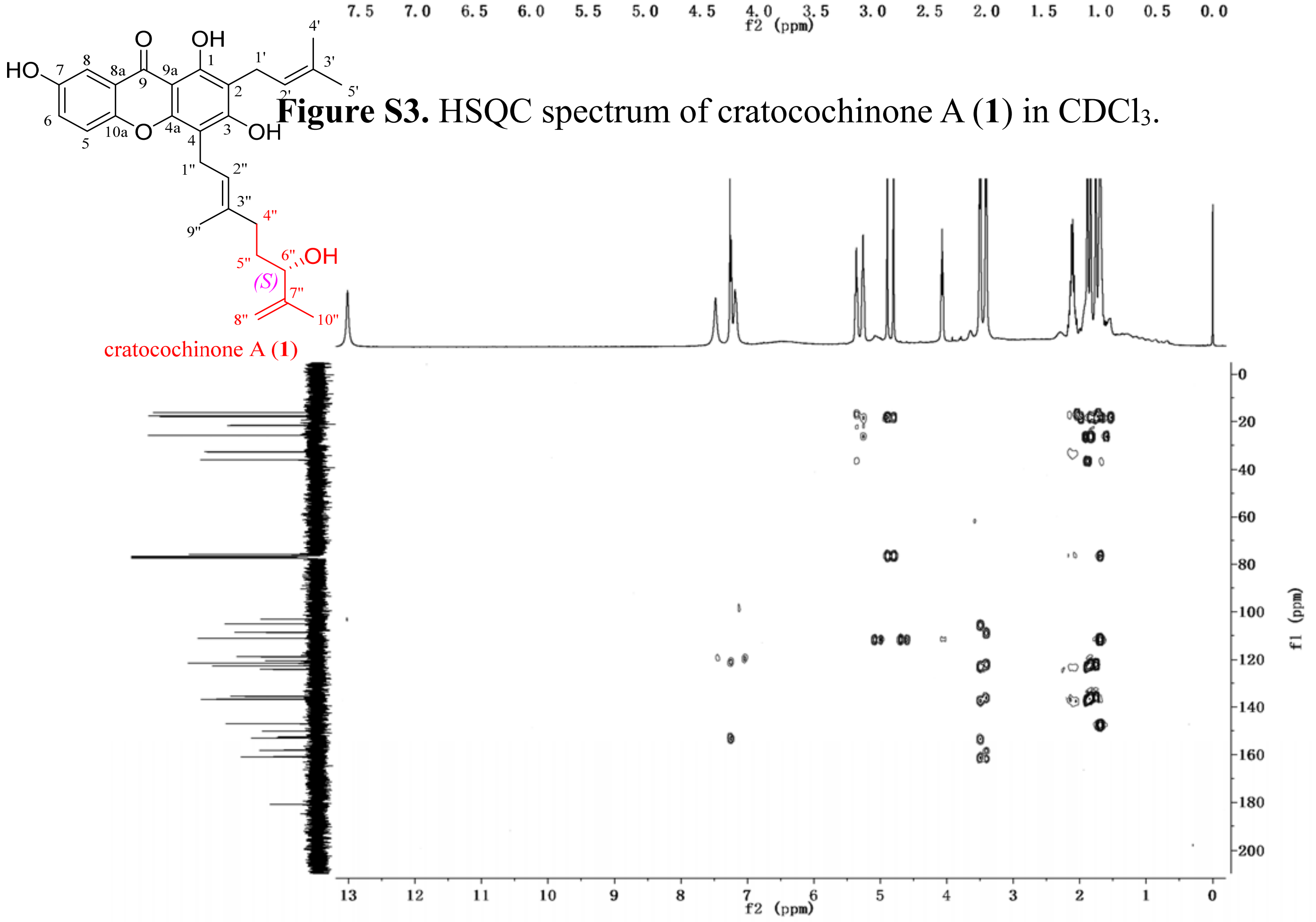

Figure S4. HMBC spectrum of cratocochinone A (**1**) in $CDCl_3$.

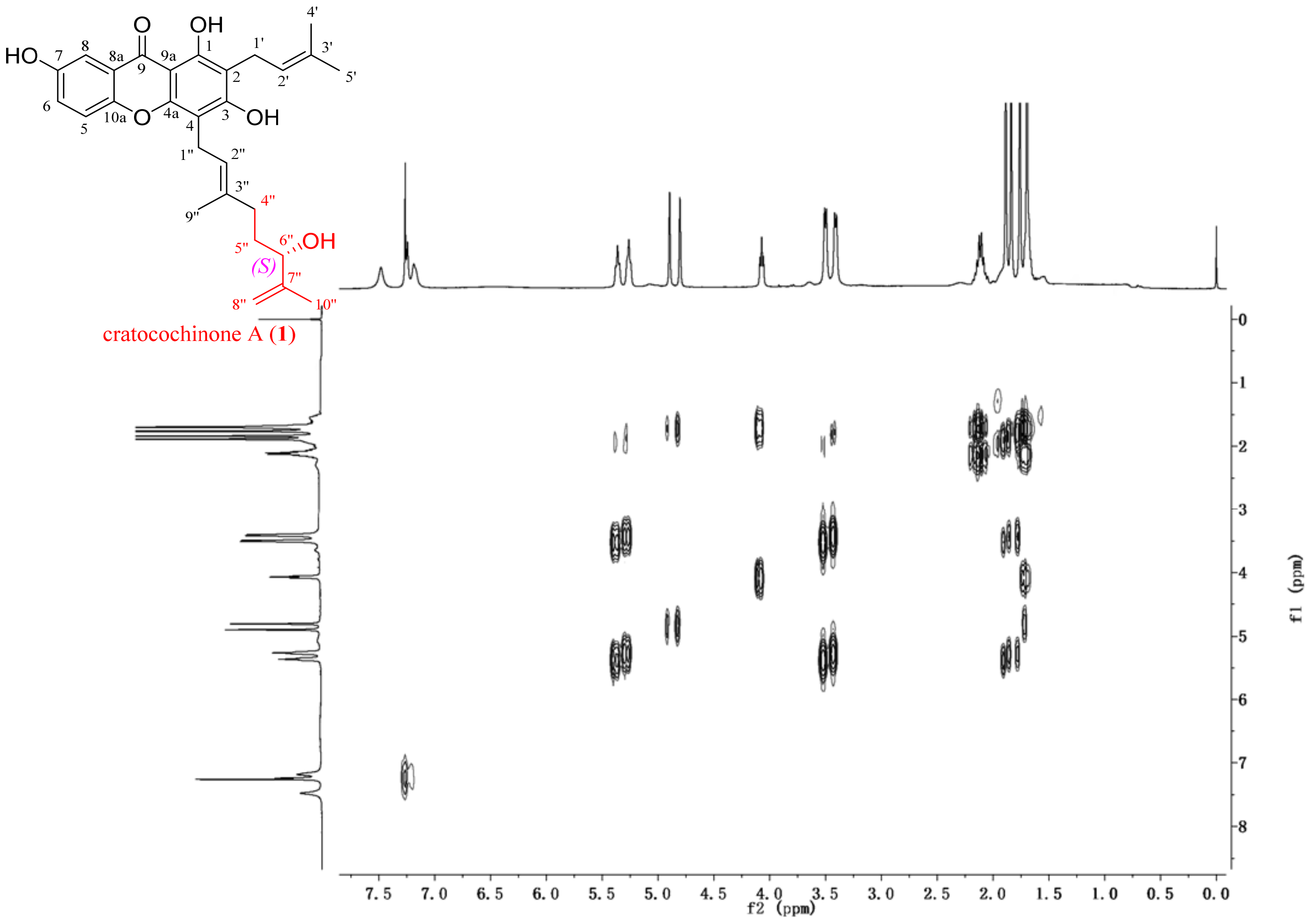

Figure S5. ^{1}H-^{1}H COSY spectrum of cratocochinone A (**1**) in $CDCl_3$.

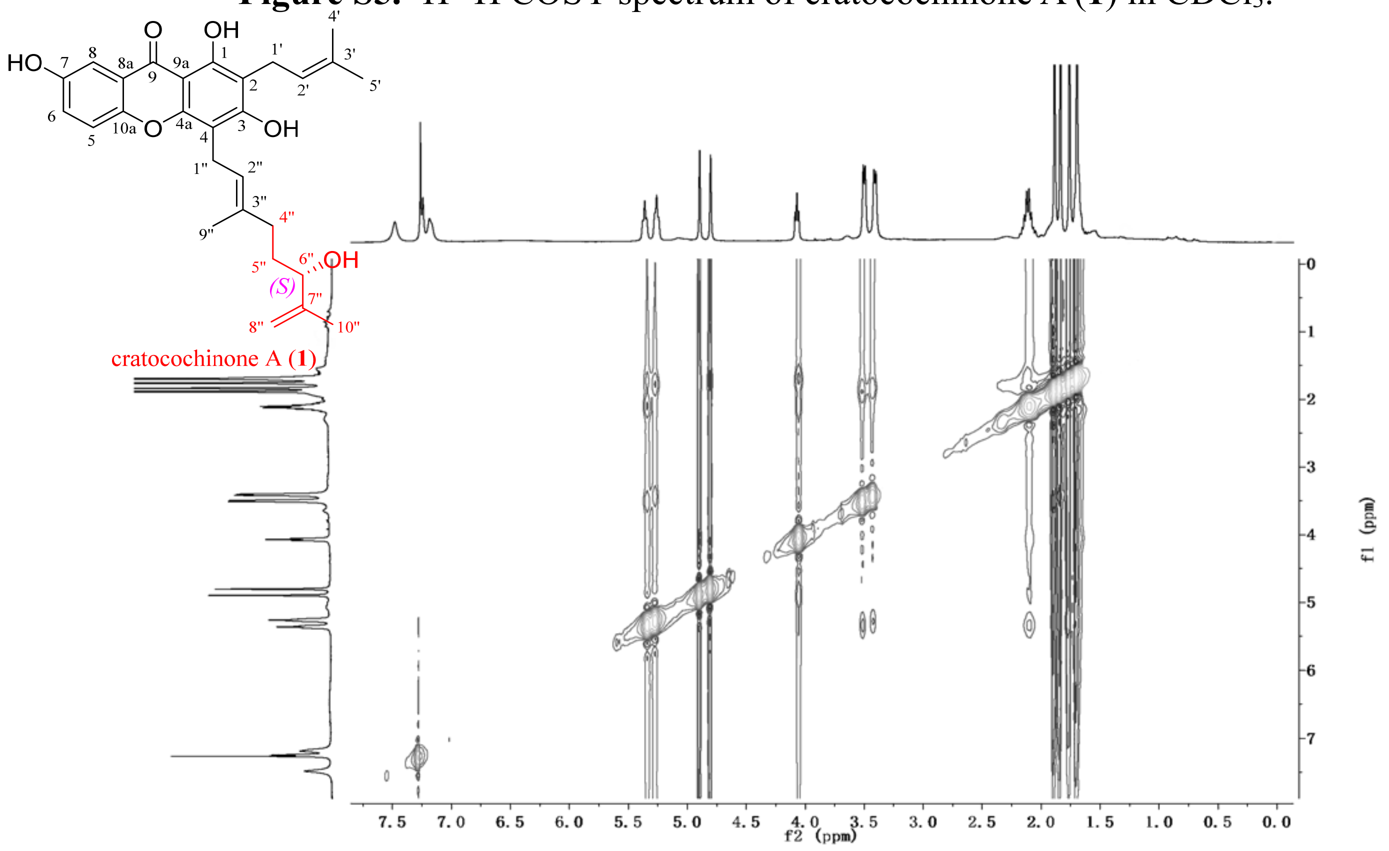

Figure S6. ROESY spectrum of cratocochinone A (**1**) in $CDCl_3$.

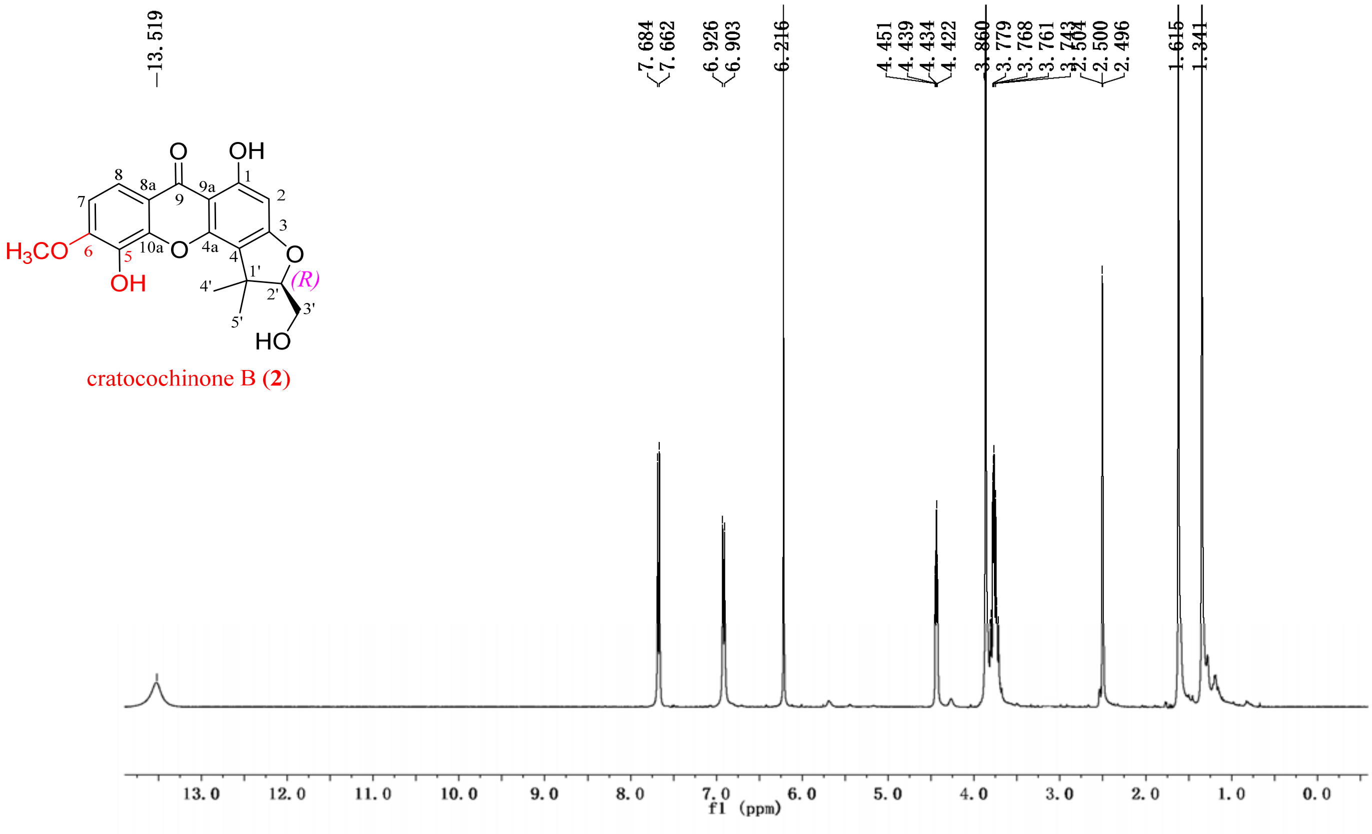

Figure S7. ^{1}H NMR spectrum of cratocochinone B (**2**) in DMSO-d_6.

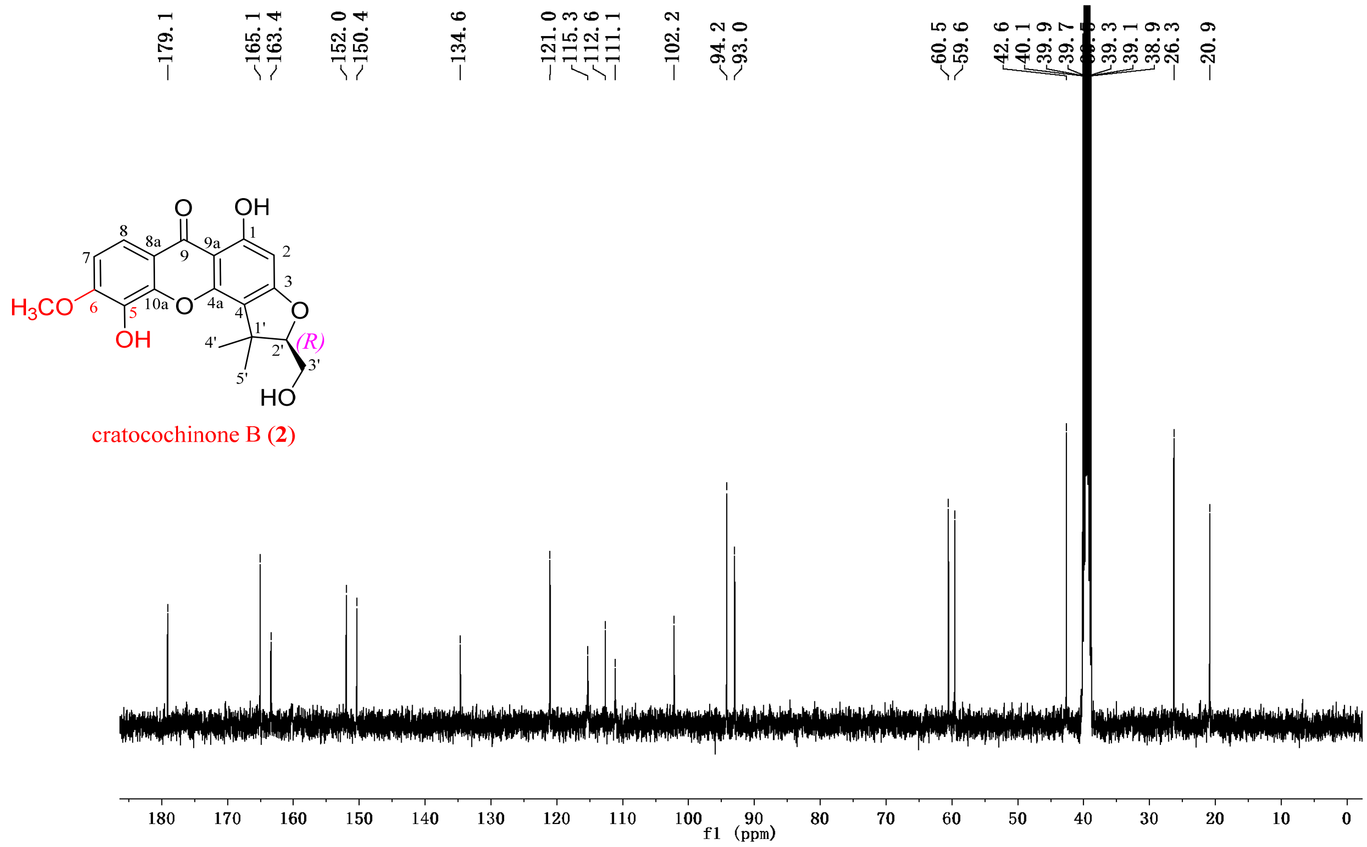

Figure S8. ^{13}C NMR spectrum of cratocochinone B (**2**) in DMSO-d_6.

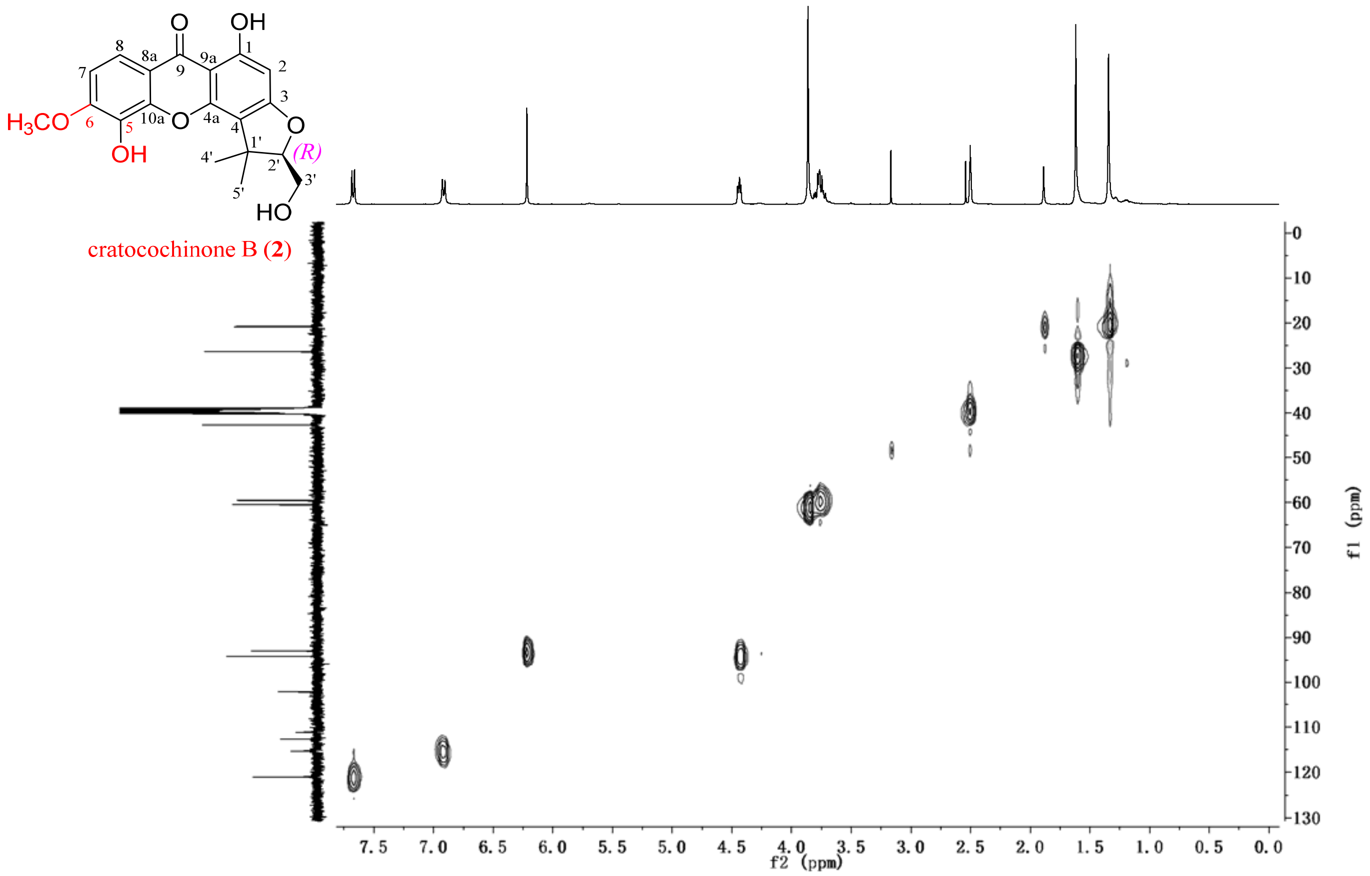

Figure S9. HSQC spectrum of cratocochinone B (**2**) in DMSO-d_6.

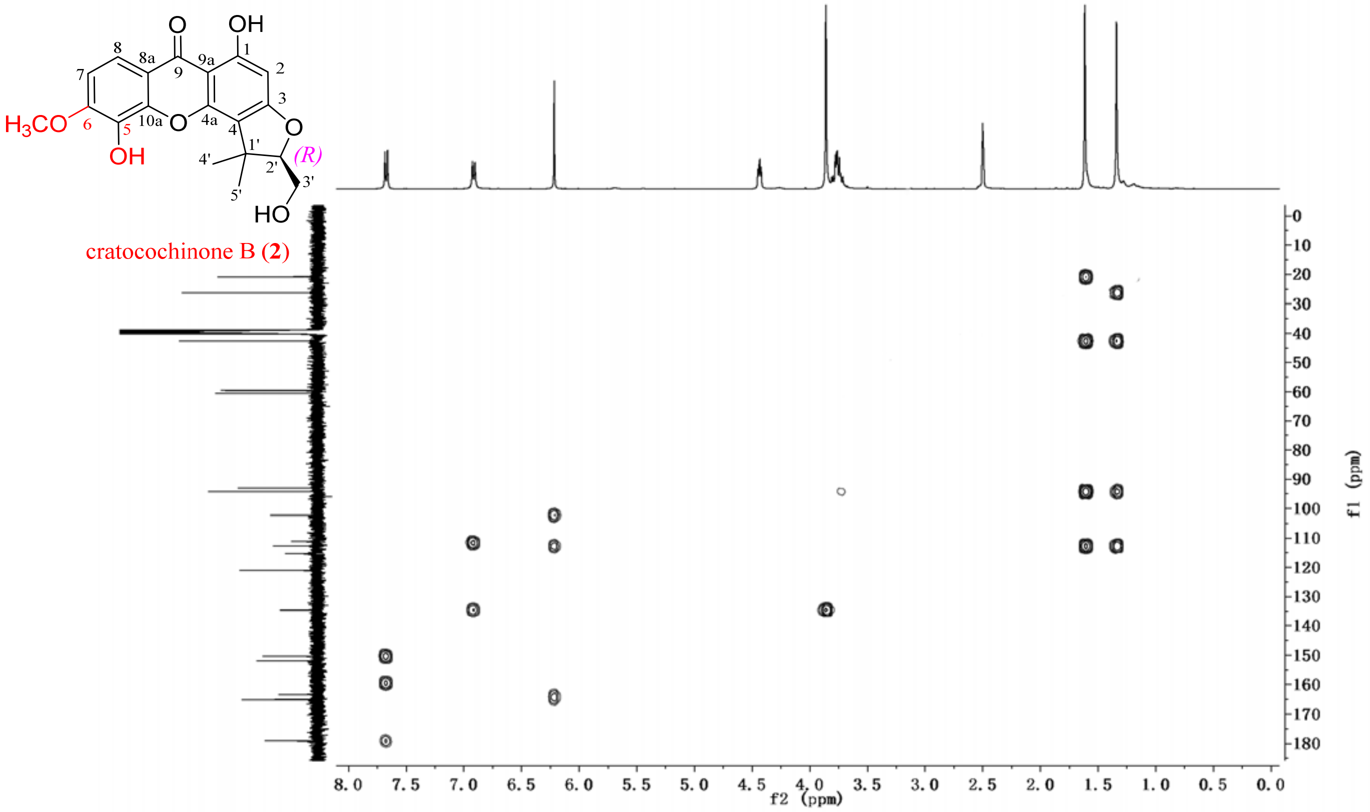

Figure S10. HMBC spectrum of cratocochinone B (**2**) in DMSO-d_6.

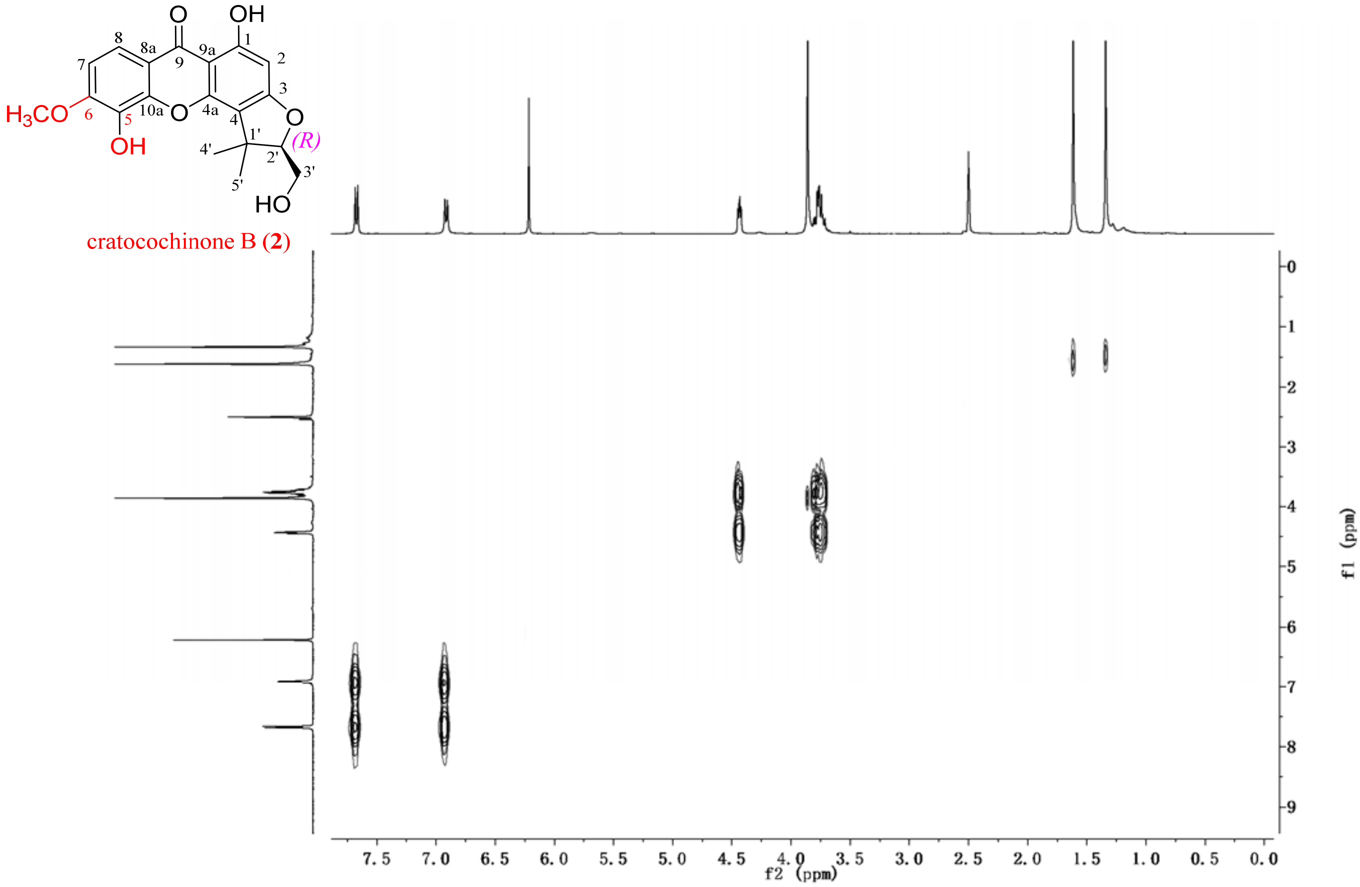

Figure S11. ^{1}H-^{1}H COSY spectrum of cratocochinone B (**2**) in DMSO-d_6.

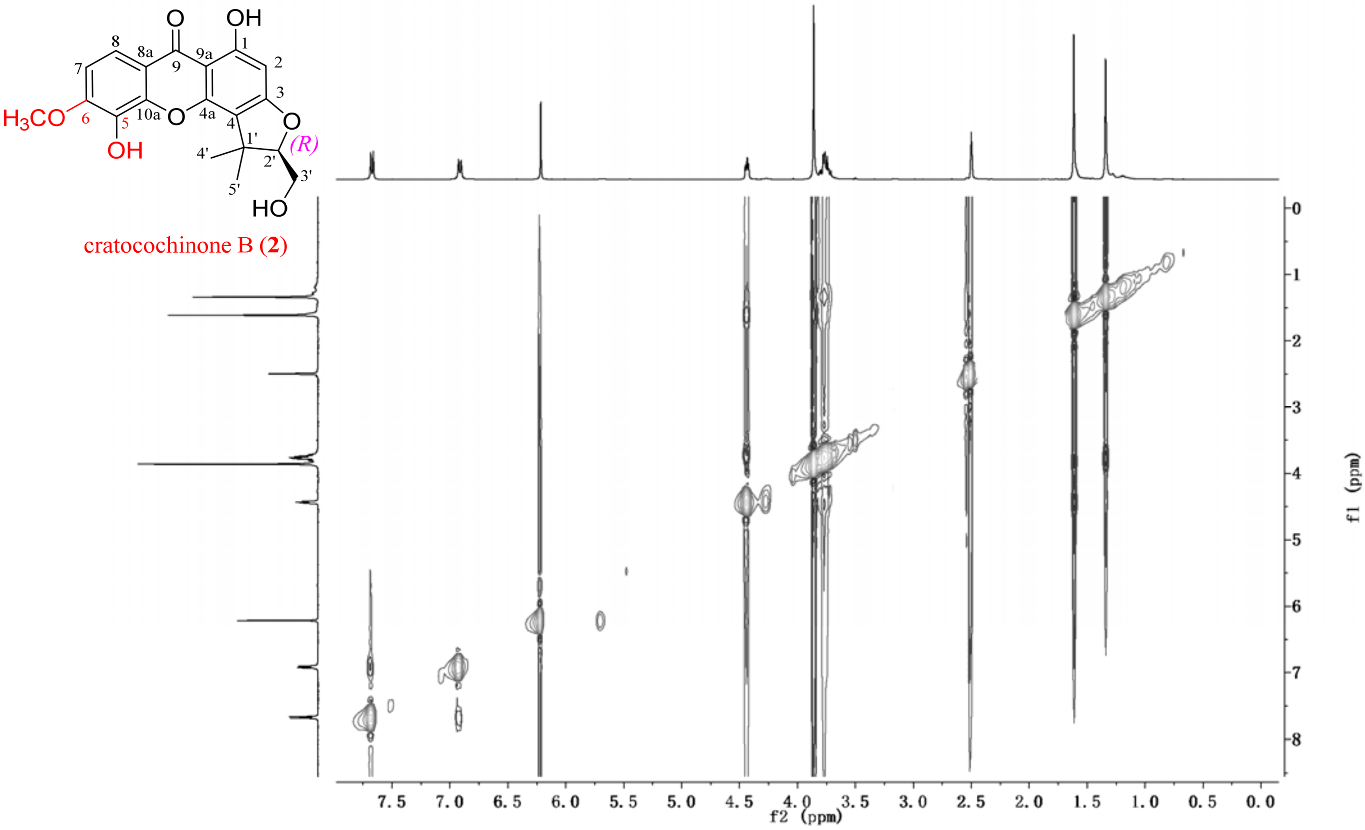

Figure S12. ROESY spectrum of cratocochinone B (**2**) in DMSO-d_6.

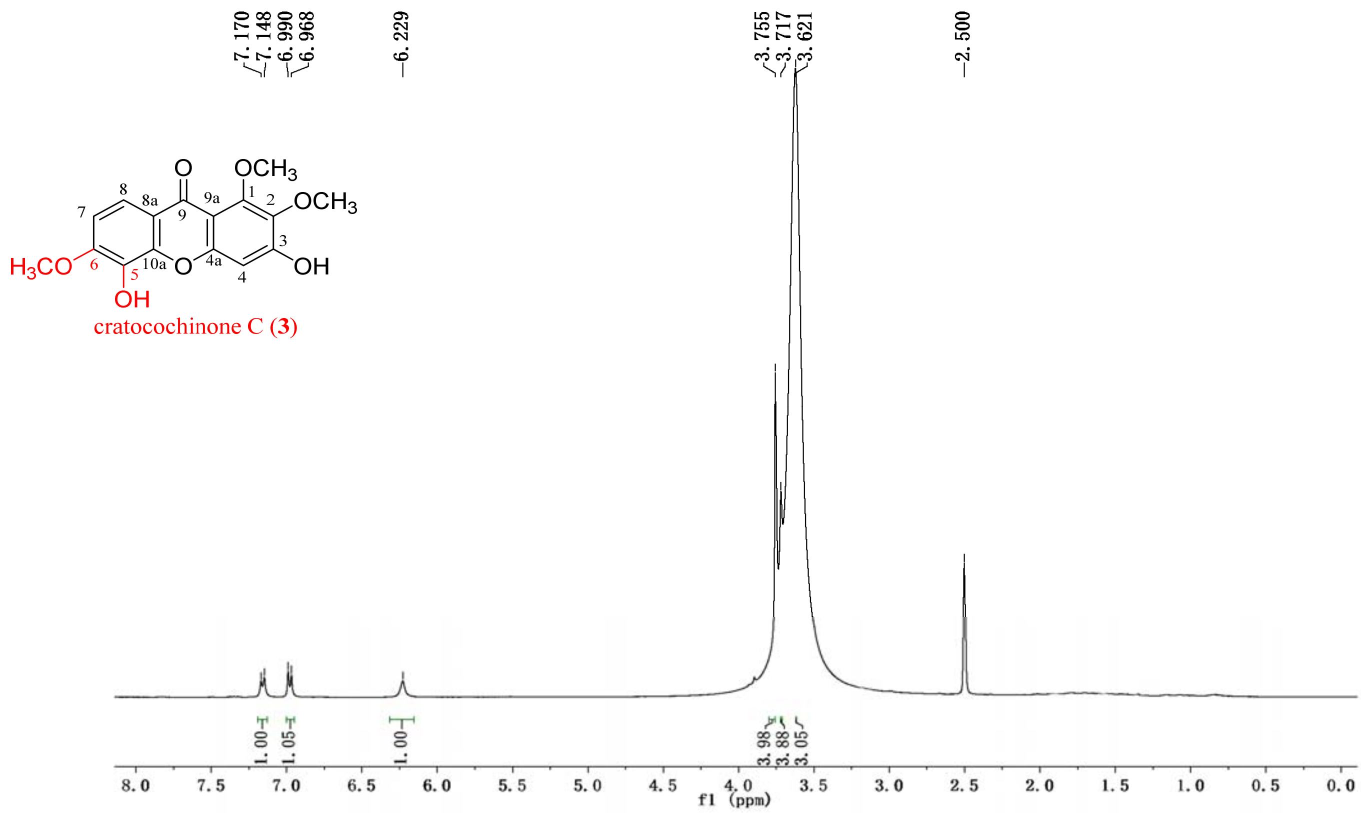

Figure S13. ^{1}H NMR spectrum of cratocochinone C (**3**) in DMSO-*d*$_6$.

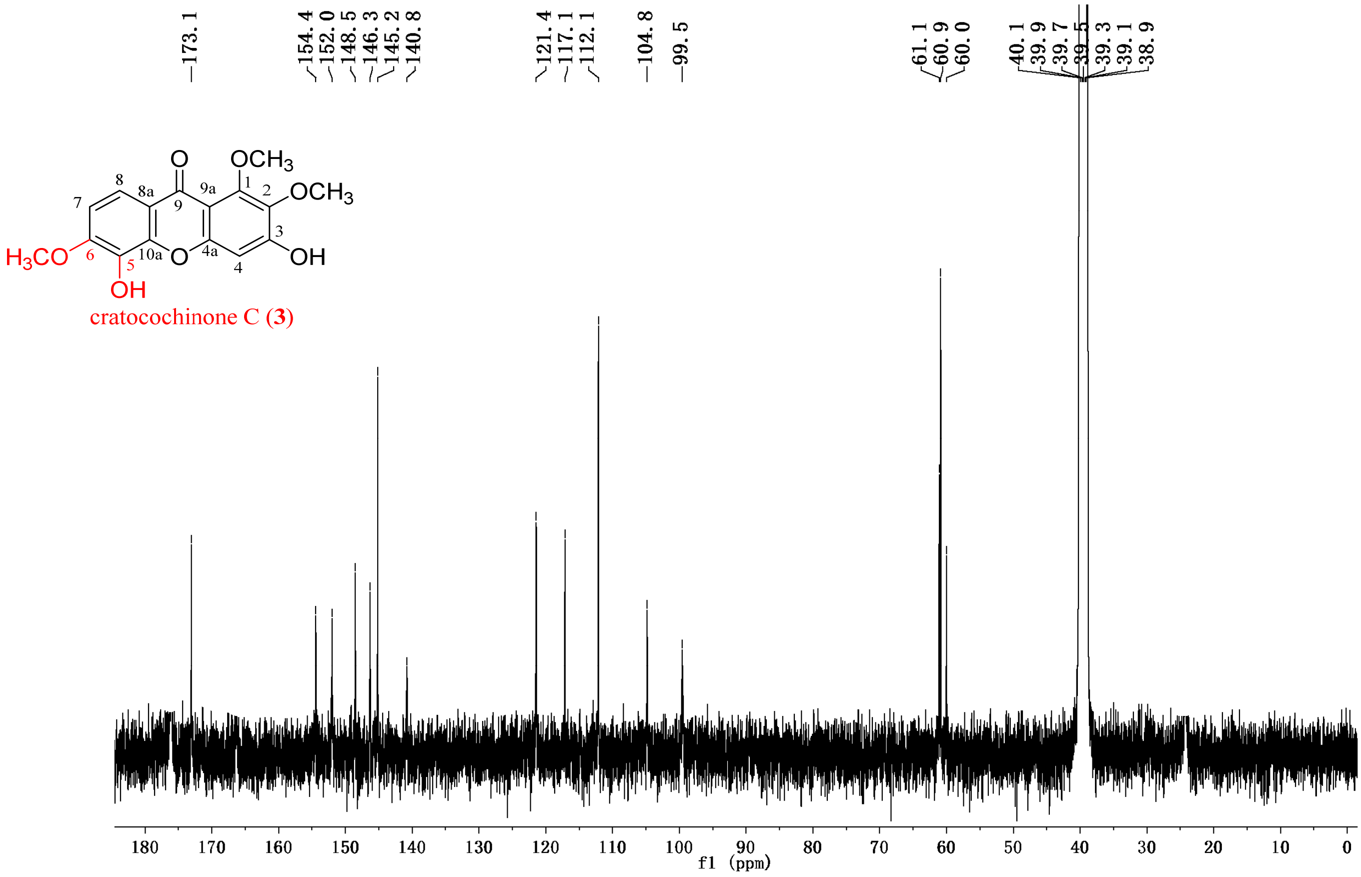

Figure S14. ^{13}C NMR spectrum of cratocochinone C (**3**) in DMSO-*d*$_6$.

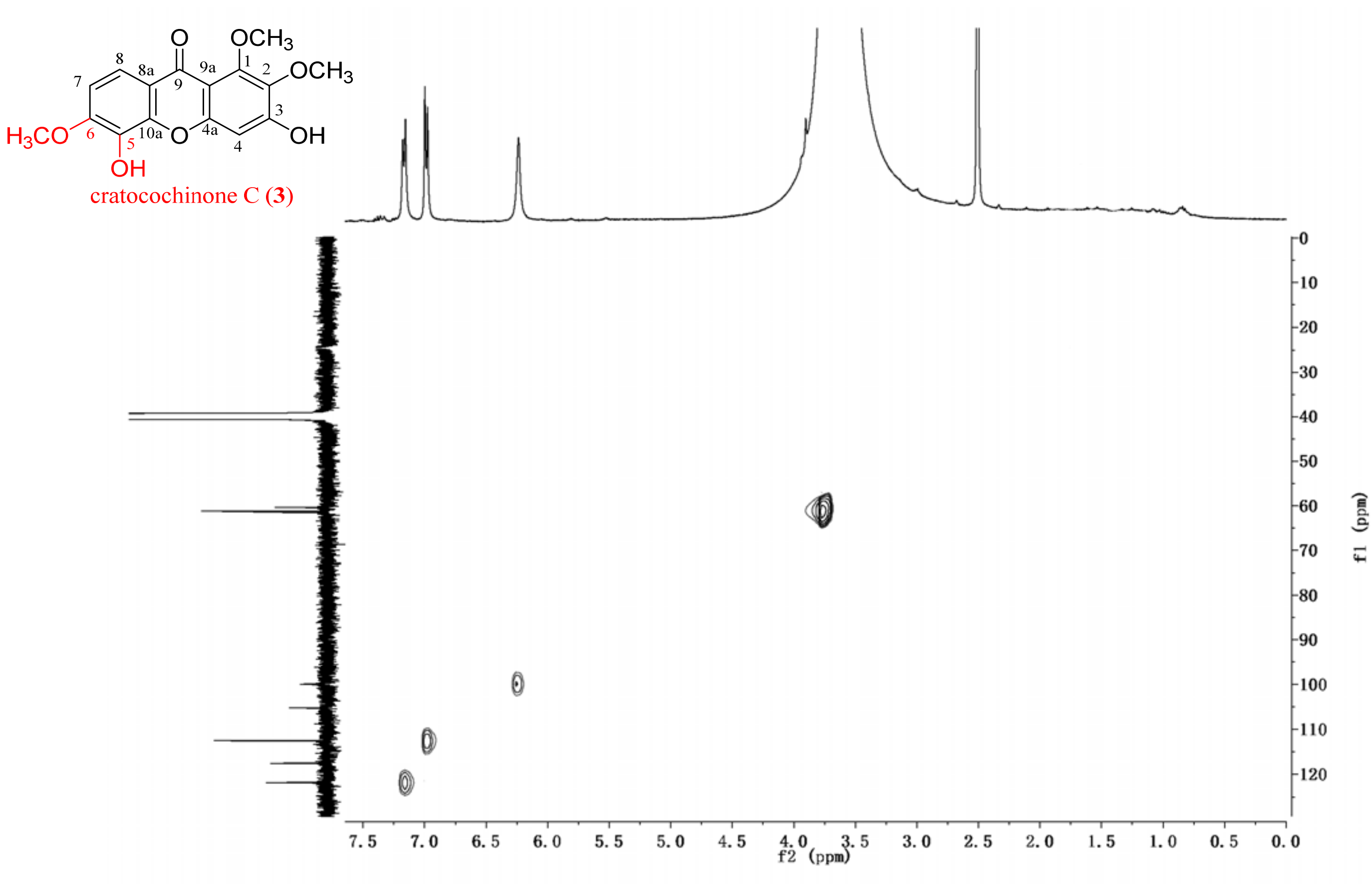

Figure S15. HSQC spectrum of cratocochinone C (**3**) in DMSO-d_6.

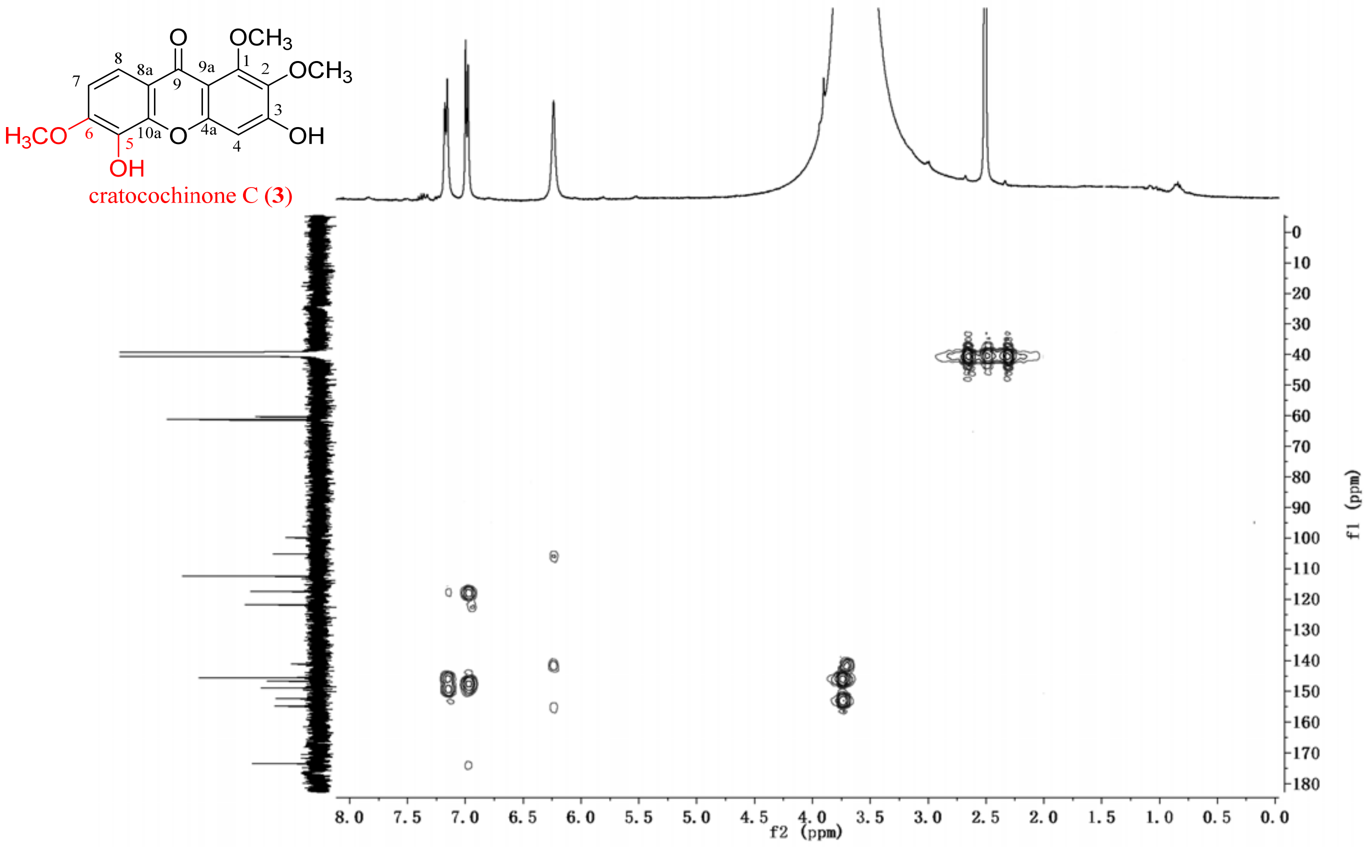

Figure S16. HMBC spectrum of cratocochinone C (**3**) in DMSO-d_6.

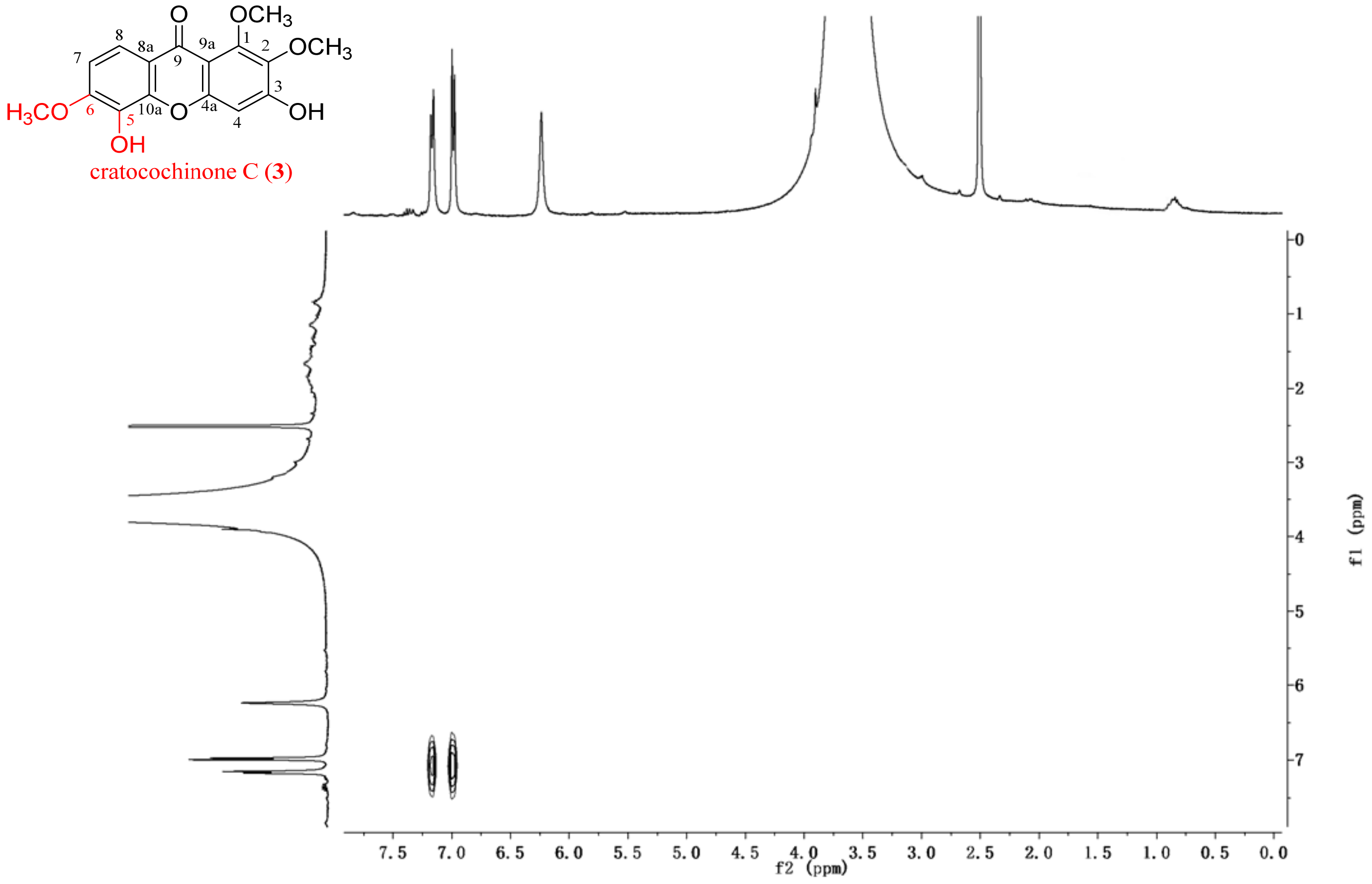

Figure S17. ^{1}H-^{1}H COSY spectrum of cratocochinone C (**3**) in DMSO-d_6.

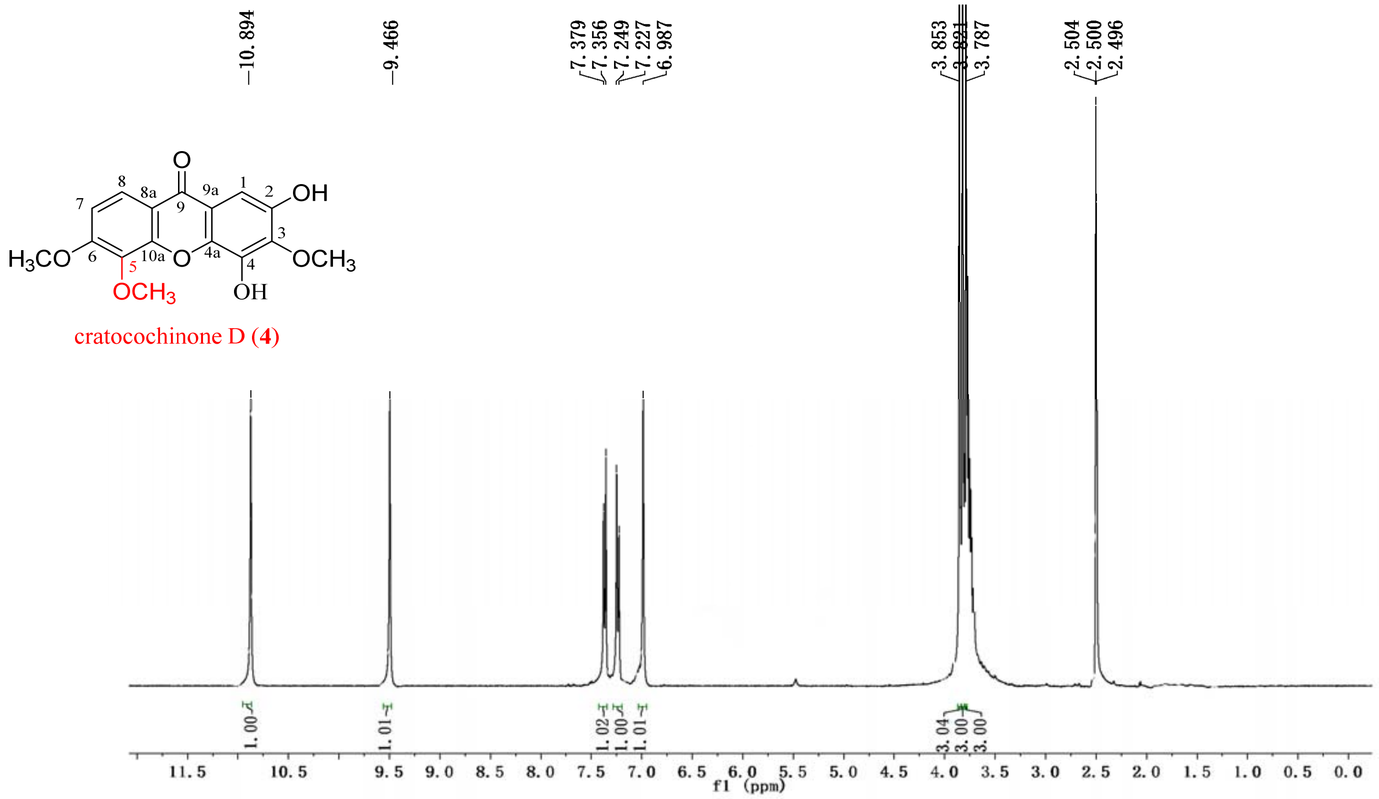

Figure S18. ^{1}H NMR spectrum of cratocochinone D (**4**) in DMSO-d_6.

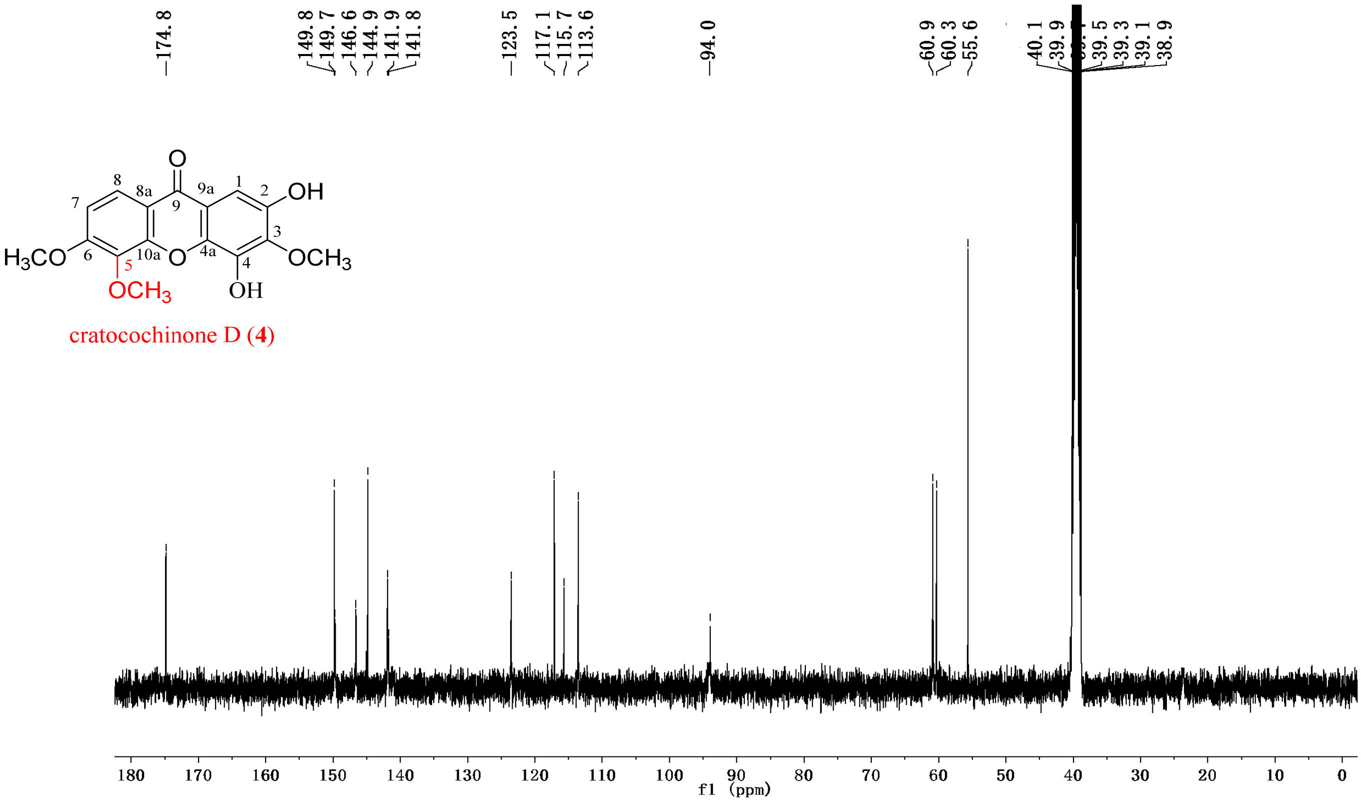

Figure S19. ^{13}C NMR spectrum of cratocochinone D (4) in DMSO-d_6.

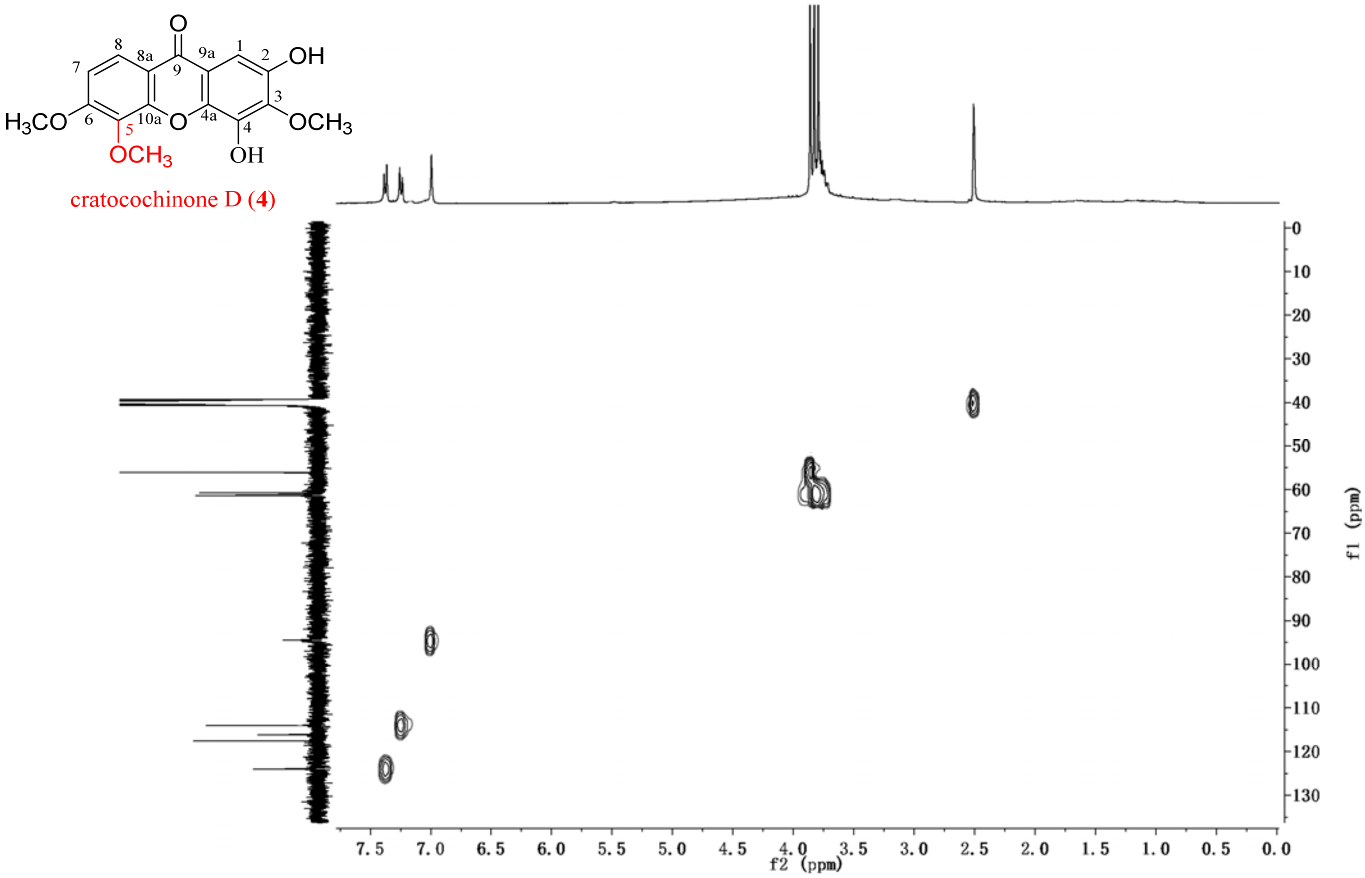

Figure S20. HSQC spectrum of cratocochinone D (**4**) in DMSO-d_6.

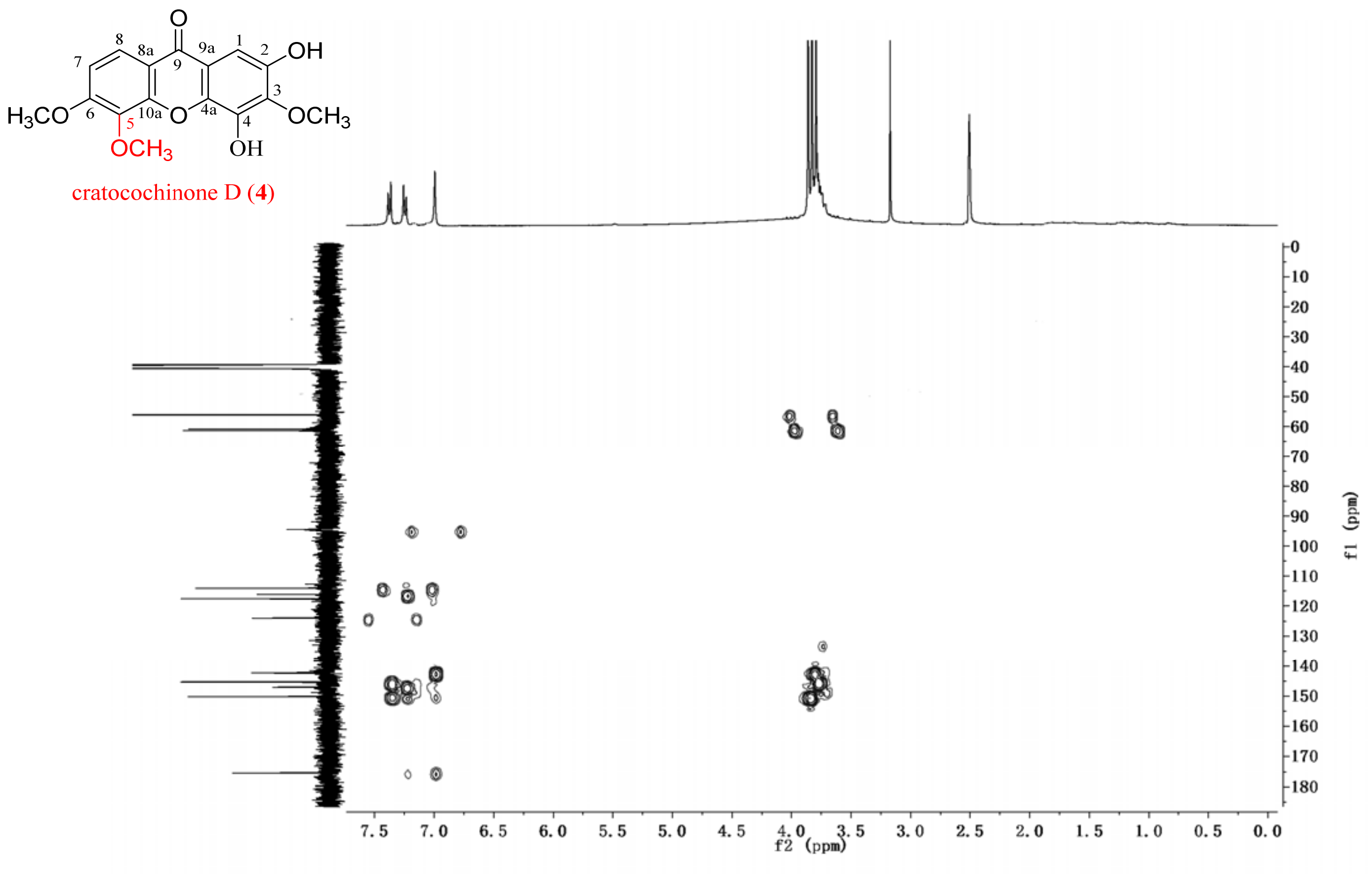

Figure S21. HMBC spectrum of cratocochinone D (**4**) in DMSO-d_6.

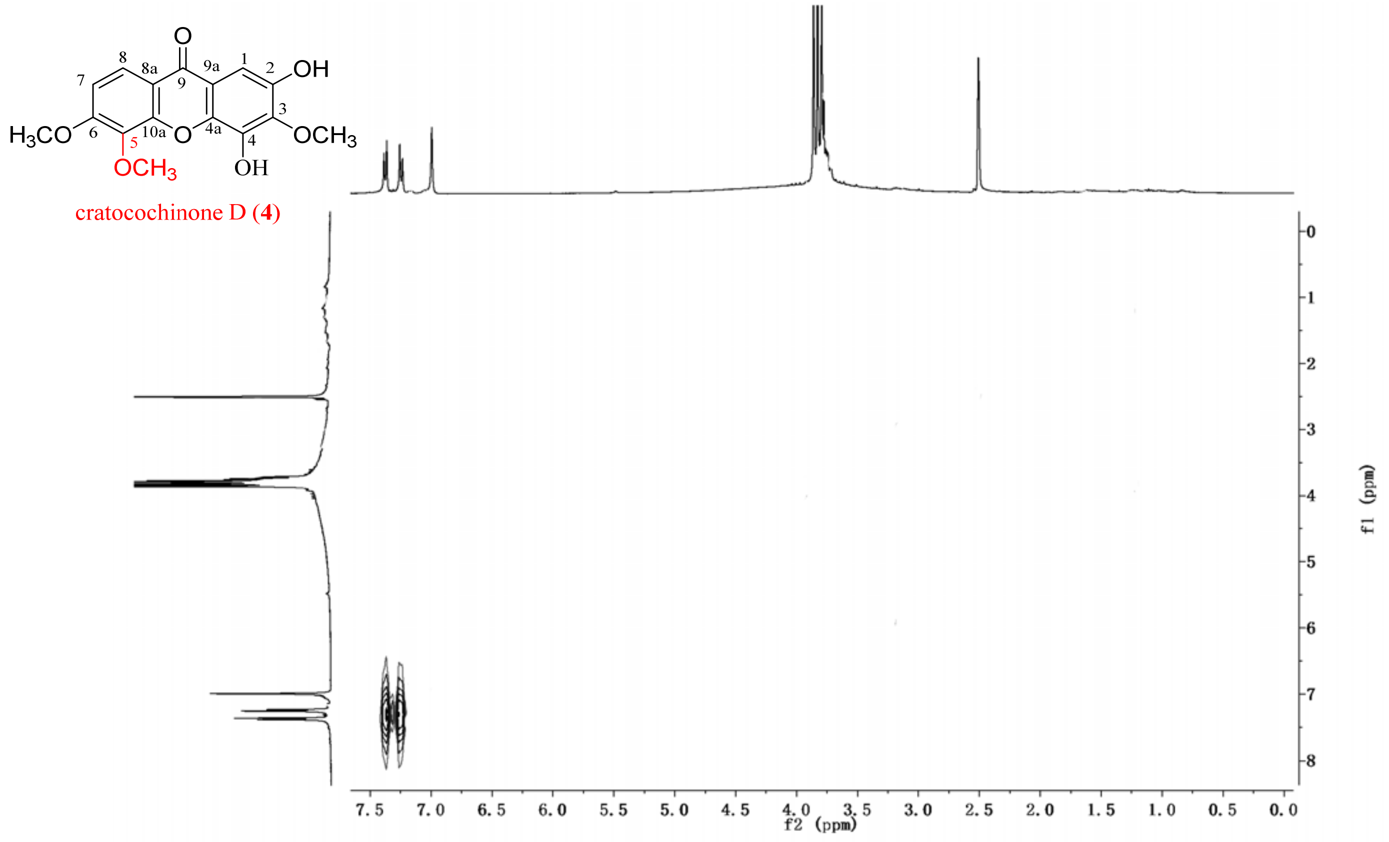

Figure S22. ^1H-^1H COSY spectrum of cratocochinone D (**4**) in DMSO-d_6.
